# Thermoadaptation in an Ancestral Diterpene Cyclase by Altered Loop Stability

**DOI:** 10.1021/acs.jpcb.1c10605

**Published:** 2022-05-18

**Authors:** David A. Hueting, Sudarsana R. Vanga, Per-Olof Syrén

**Affiliations:** † School of Engineering Sciences in Chemistry, Biotechnology and Health, Science for Life Laboratory, KTH Royal Institute of Technology, Stockholm 114 28, Sweden; ‡ School of Engineering Sciences in Chemistry, Biotechnology and Health, Department of Fibre and Polymer Technology, KTH Royal Institute of Technology, Stockholm 114 28, Sweden

## Abstract

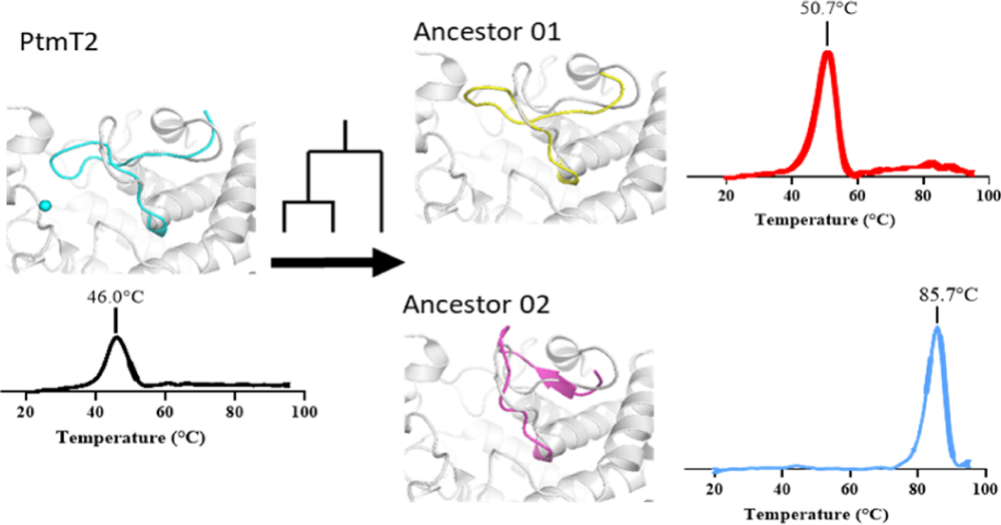

Thermostability is the key to maintain the structural integrity and catalytic activity of enzymes in industrial biotechnological processes, such as terpene cyclase-mediated generation of medicines, chiral synthons, and fine chemicals. However, affording a large increase in the thermostability of enzymes through site-directed protein engineering techniques can constitute a challenge. In this paper, we used ancestral sequence reconstruction to create a hyperstable variant of the *ent*-copalyl diphosphate synthase PtmT2, a terpene cyclase involved in the assembly of antibiotics. Molecular dynamics simulations on the μs timescale were performed to shed light on possible molecular mechanisms contributing to activity at an elevated temperature and the large 40 °C increase in melting temperature observed for an ancestral variant of PtmT2. *In silico* analysis revealed key differences in the flexibility of a loop capping the active site, between extant and ancestral proteins. For the modern enzyme, the loop collapses into the active site at elevated temperatures, thus preventing biocatalysis, whereas the loop remains in a productive conformation both at ambient and high temperatures in the ancestral variant. Restoring a Pro loop residue introduced in the ancestral variant to the corresponding Gly observed in the extant protein led to reduced catalytic activity at high temperatures, with only moderate effects on the melting temperature, supporting the importance of the flexibility of the capping loop in thermoadaptation. Conversely, the inverse Gly to Pro loop mutation in the modern enzyme resulted in a 3-fold increase in the catalytic rate. Despite an overall decrease in maximal activity of ancestor compared to wild type, its increased thermostability provides a robust backbone amenable for further enzyme engineering. Our work cements the importance of loops in enzyme catalysis and provides a molecular mechanism contributing to thermoadaptation in an ancestral enzyme.

## Introduction

Enzyme thermostability is the key to enable extended catalytic versatility of enzymes for synthetic biology applications.^1−4^ Therefore, being able to engineer enzymes with increased thermostability while maintaining high catalytic activity is desirable.^5,6^ Ancestral sequence reconstruction is a rapidly emerging bioinformatics method to generate sequences of putative ancestral enzymes and proteins, that according to evolutionary models should be thermostable, as protein robustness is the key for evolvability by tolerating additional mutations.^7,8^ In ancestral sequence reconstruction,^9,10^ a phylogenetic tree is constructed from extant protein sequences and from which presumed ancestral sequences can be retrieved.

Conserved sequence regions in a protein family of interest can contribute to enhanced thermostability by so-called consensus effects.^11^ However, the molecular mechanisms underpinning thermoadaptation of enzyme catalysis remain incompletely understood.^12−14^ Possible contributions to increased temperature stability stem from enhanced structural rigidity and altered protein dynamics.^1^ In particular, the importance of flexibility of loops for protein function and enzyme catalysis has gained increased attention.^15^ For some enzymes such as terpene cyclases,^16^ loop dynamics is a necessity for expedient catalysis by capping the active site following substrate binding, shielding reactive intermediates from the solvent, and enabling efficient transition-state stabilization.^16^ Proteins with flexible loops have previously been shown to exhibit allokairic regulation.^17,18^ Herein, we used ancestral sequence reconstruction in concert with enzyme kinetics and molecular dynamics (MD) simulations to study how the flexibility of a loop surrounding the active site in ancestral and modern terpene cyclases is linked to the catalytic activity at different temperatures.

**Scheme 1:**
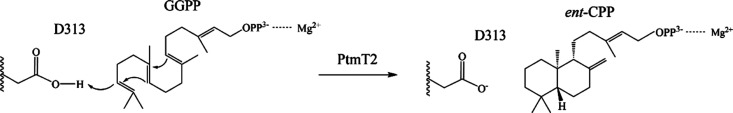
Cyclization of GGPP to *ent*-CPP Catalyzed by PtmT2 The catalytic aspartate is shown.

Terpenes and terpenoids are an abundantly present class of natural products with potent biological activities. There is a plethora of known terpenes based on the same simple formula (C_5_H_8_)_*n*_ with a large variety of potent biological functions and applications, including steroids, antibiotics, fine chemicals, flavors, and fragrances.^16^ Biosynthesis of terpenes is founded on the initial elongation of simple C_5_ linear building blocks starting from dimethylallyl diphosphate, which upon ionization reacts with isopentenyl diphosphate to generate C_10_ geranyl diphosphate (GPP), which can then be further elongated.^19^ Cyclization of linear polyisoprenes by terpene cyclase enzymes *via* complex electrophilic carbocationic cascades is the key for terpene diversity.^16^*Ent*-copalyl diphosphate synthase (PtmT2) from *Streptomyces platensis* is a bacterial class II diterpene cyclase that utilizes C_20_ geranylgeranyl diphosphate (GGPP) as substrate to generate cyclic *ent*-copalyl diphosphate^20^ (*ent*-CPP, Scheme 1) *en route* to forge the antibiotics platensimycin and platencin.^21^ Its X-ray crystal structure is known,^20^ facilitating structure–functional studies.

Terpene cyclases are classified into two main groups: class I enzymes initiate cyclization by cleaving off the terminal pyrophosphate, whereas class II terpene cyclases rely on protonation of a terminal isoprene/oxirane. The difference in the active site architecture between class I and class II enzymes are found in amino acid motifs involved in metal binding (DDxxD) and protonation (DxDD), respectively. Specific aromatic side chains are important to stabilize carbocations formed during the different cyclization events.^22,23^ PtmT2 is an interesting class II terpene cyclase in converting a phosphorylated substrate otherwise characteristic of class I enzymes, leaving the pyrophosphate group intact attached to the cyclic core formed, and in this way generating a potent substrate for class I terpene cyclases. The protein anchors the substrate pyrophosphate through the binding of one Mg^2+^ mediated by the D^128^xxxxE^133^ motif.^20^

In this research, we used ancestral sequence reconstruction to generate a hyperstable terpene cyclase utilizing the sequence of extant PtmT2 as a template. MD simulations on the microsecond timescale showed that protein compaction essentially remained unchanged, when going from modern to ancestral enzyme, whereas the conformation and flexibility of a loop capping the active site differed. This work corroborates ancestral sequence reconstruction as a useful tool in protein engineering and design and provides molecular insights into thermoadaptation of biocatalysts.

## Materials and Methods

### Ancestral Sequence Reconstruction

The sequences used for phylogenetic analyses were found *via* a homology search based on the *ent*-copalyl diphosphate synthase from *S. platensis* (PtmT2, GenBank: A0A023VSF1). The 250 sequences with the highest similarity were selected and aligned *via* MEGA-X^24^ using the MUSCLE algorithm.^25^ The alignment was improved by deleting duplicates and manual trimming of sequences. The phylogeny of the aligned sequences was constructed using MEGA-X and subsequently confirmed with IQTree.^26^ The best evolutionary model for reconstruction was found to be LG + F + G + I.^27^ The tree was tested using the bootstrap method with 1000 bootstrap replications. The ancestral sequences were reconstructed using the ancestral sequence inference option in MEGA-X. The initial five amino acids of the extant protein were introduced, replacing the predicted ancestral initial amino acids to promote expression. The sequences were then codon-optimized for expression in *Escherichia coli*. The genes used in this study were purchased from GeneArt Services (ThermoFisher Scientific, USA) equipped with an N-terminal His_6_-tag.

### Protein Expression and Purification

The sequences were cloned into pET22b(+) and transformed into chemically competent C43(DE3) cells. Cells were plated onto nutrient agar plates supplemented with 100 μg/mL ampicillin and incubated overnight at 37 °C at 200 rpm. Colonies from the plate were used to inoculate 3 mL 2-YT medium (16 g L^–1^ tryptone, 10 g L^–1^ yeast extract, and 5 g L^–1^ NaCl) with 100 μg/mL ampicillin and the pre-culture was incubated at 37 °C at 200 rpm overnight. The cells were propagated into 100 mL 2-YT medium supplemented with 100 μg/mL ampicillin and grown at 37 °C at 200 rpm until OD_600_ reached 0.6–0.8. The cells were induced with 1 mM final concentration of isopropyl β-d-1-thiogalactopyranoside and protein expression was performed at 18 °C at 180 rpm overnight. The cells were harvested by centrifugation at 3800×*g* at 4 °C. The supernatant was discarded, and 50 mL of culture cell pellets were stored at −20 °C.

The frozen cell pellets were lysed with B-PER Complete Bacterial Protein Extraction Reagent (ThermoFisher Scientific, USA) supplemented with 20 mM imidazole and left at 25 °C for 15 min at 180 rpm in a cultivation shaker. The lysate was spun down at 3800×*g* at 4 °C for 25 min. The supernatant was transferred to a 15 mL falcon tube to which 500 μL of equilibrated Ni-NTA agarose (Qiagen, Germany) was added. The supernatant and the beads were incubated for 2 h at 4 °C on a shaking block. The suspension was centrifuged at 380×*g* for 1 min and the supernatant was discarded. The beads were washed three times by centrifugation at 380×*g* for 1 min at 4 °C in 3 mL wash buffer (50 mM tris(hydroxymethyl)aminomethane (Tris)-HCl, 500 mM NaCl, 20 mM imidazole, and at pH = 7.4). After washing, the protein was eluted by the addition of 3 mL elution buffer (50 mM Tris-HCl, 500 mM NaCl, 300 mM imidazole, pH 7.4), at 4 °C. After incubation for 2 min, the supernatant was extracted through centrifugation at 380×*g* for 2 min at 4 °C. The supernatant with protein was desalted with disposable PD 10 columns (Cytiva, USA) into storage buffer (50 mM Tris-HCl, 100 mM NaCl, 50 mM KCl, and at pH = 7.4). The protein was concentrated by transferring the supernatant to an Amicon Ultra-15 Centrifugal Filter (30 kDa cutoff) and by spinning for 25 min at 3800×*g* at 4 °C. The protein concentration was measured using an Implen NanoPhotometer NP80 (Germany) at 280 nm using a molecular weight of 55498 Da and an extinction coefficient of 97,860 (M^–1^ cm^–1^). The purity of the protein was then verified by SDS–PAGE using a 4–15% Mini-PROTEAN TGX Precast Protein Gel (Bio-Rad). The protein was stored at 4 °C (for a maximum of 1 week).

### Thermostability

The thermostability of the proteins was measured by nano differential scanning fluorimetry using a Prometheus NT.48 nanoDSF instrument (NanoTemper Technologies, Germany). The protein stocks were diluted to a concentration of 2 mg/mL and were introduced into a glass capillary through capillary force. The unfolding of the proteins was measured by the ratio of fluorescence at 330 and 350 nm. Unfolding of the proteins was recorded between 20 to 95 °C with 1 °C/min increments. By monitoring the ratio between the intensity of 330 and 350 nm and analyzing the derivative of the change, the melting points were determined at the maximum of the derivative.

### Enzyme Activity

The enzyme activity is determined through an analysis of the reaction rate of the biocatalysts at temperatures ranging from 30 to 90 °C. The GGPP concentration during all reactions was kept constant at 50 μM (unless written otherwise). The reaction was prepared in an Eppendorf tube with 167.6 μL of reaction buffer (50 mM citric acid, pH = 6.0, 1 mM MgCl_2,_ 1 mM β-mercaptoethanol, and 10% glycerol) and 4.4 μL stock of geranylgeranyl diphosphate ammonium salt (Merck, USA). The enzyme concentrations used in these experiments for PtmT2, Anc01, and Anc02 are 50, 50, and 250 nM, respectively. The total reaction volume was 176 μL. The reaction was pre-incubated in a thermomixer (Eppendorf, Germany) at the respective temperatures for 10 min after which the protein was added. The reaction was left at the respective temperatures at 900 rpm shaking speed. 50 μL samples were taken from the reaction tube and quenched with 50 μL methanol. The sample was centrifuged for 1 min at 9000×*g* after which the sample was analyzed by HPLC–MS. Additionally, as controls, samples containing only buffer, buffer and enzyme, buffer and substrate, and a timepoint zero sample were prepared and analyzed.

### Mutagenesis

Mutagenesis was performed with the Q5 Site-Directed Mutagenesis Kit Protocol (New England Biolabs, USA). The protocol from the supplier was followed. 12.5 μL Q5 Hot Start High-Fidelity 2X Master Mix was mixed with a final concentration of 0.5 μM forward, 0.5 μM reverse primer (primer sequences given in Supporting Information, Table S1), and 25 ng template DNA to a final volume of 25 μL. Thermocycling was according to the following PCR protocol: 98 °C for 10 s, 61 °C for 30 s, 72 °C for 3.5 min. The PCR product was digested with DpnI and then transformed into XL1-Blue competent cells *via* heat shock.

### HPLC

The samples were run on an HPLC–MS system (Agilent Technologies, USA) over an XBridge C18 3.5 μm, 3.0 × 50 mm column. The protein was eluted with an acetonitrile/10 mM NH_4_HCO_3_ buffer (pH = 10) gradient, from 10 to 97% acetonitrile over 3 min. The flow was 1 mL/min and 1 min equilibration time before injection was used with the column oven temperature set at 40 °C. GGPP and *ent*-CPP were measured at 210 nm wavelength and analyzed through the internal peak integration of the provided Agilent software.

## Computational Approaches

### Homology Modeling of Ancestors

The crystal structure of the PtmT2 was retrieved from the Protein Data Bank (PDB ID: 5BP8,^20^) and the three-dimensional structures of the ancestors were predicted by YASARA^28,29^ homology modeling program (Version 20.8.23). We have used 3 PsiBLAST iterations per sequence with a maximum allowed *E*-value per template of 0.1. A total number of templates were chosen to be 5; 50 loop conformations were tried; and 10 residues at the terminal was allowed to be added. The sequence similarity between the template PDB ID: 5BP8 (extant PtmT2) and Anc01 and Anc02 are 97.9 and 74.6%, respectively. To ensure the quality of the obtained homology structures, corresponding ψ and ϕ angles of their Ramachandran plots were assessed, as well as the use of YASARA’s inbuilt scoring function, *Z*-score. The overall *Z*-scores for all models have been calculated as the weighted averages of the individual components using the formula
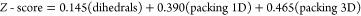


The overall score thus captures the correctness of backbone- (Ramachandran plot) and side-chain dihedrals, as well as packing interactions.

### Mg^2+^ Binding Site Modeling

It was postulated that the metal-binding site in PtmT2 contains a single Mg^2+^;^20^ however, the catalytically important metal-binding site in PtmT2 remains elusive even after extensive computational, structural, and mutagenesis studies.^30−32^ The metal-binding site in PtmT2 is expected to be at the active site entrance to anchor the substrate diphosphate group and stabilize the pre-folded substrate conformation by enzyme–metal–substrate interactions.^20^ Accurately identifying the metal-binding site is essential for both predicting and understanding the protein structure and function. The Mg^2+^ binding site in PtmT2 was predicted using the metal ion-binding (MIB) site prediction and docking server,^33,34^ which employs the fragment transformation method. This fragment transformation method tries to structurally align query protein fragments with metal ion-binding residue templates obtained from the PDB. The sequence and structural similarity of each residue in these alignments are used to provide a score. Residues that score above the assigned alignment-score threshold are predicted to bind metal ions and the top ranked metal-bound structures were selected for further computational approaches.

### Molecular Dynamics Simulations

MD simulations were performed using GROMACS 2019.3^35,36^ using Amber 99sb-ildn force field^37^ containing updated backbone ψ and ϕ angles and side-chain torsions. Protein structure was solvated with SPC/E^38^ water molecules centered in a periodic box extended by 1 nm from the protein edges. An appropriate number of water molecules were randomly replaced with Na^+^ and Cl^–^ counterions to keep the system neutral (at pH = 7) and at a physiological concentration using the genion^36,39^ module in GROMACS. Long-range electrostatic interactions were treated with Particle mesh Ewald method with a cutoff of 1 nm for both electrostatics and van der Waals interactions. Energy minimization was carried out for 50 ps using the steepest descent algorithm. After performing energy minimization, the structures were equilibrated for 100 ps at two different temperatures (303 and 343 K) by position restrains to maintain pressure (1 bar) and temperature while relaxing the solvent. Following equilibration, both systems were subjected to 1 microsecond final MD simulation under the same conditions. Step size was set to 2 fs using the LINCS algorithm^40^ for constraining bonds and the leapfrog algorithm^41^ for integration. All the simulations were replicated three times with different random seeds.

### Analysis and Visualization

The root mean square deviation (RMSD) values and root mean square fluctuations (RMSF) were calculated using GROMACS embedded tools. These were based on standard RMSD and RMSF calculations following rigid body translation and rotation for structural superposition in Cartesian space, minimizing the resulting values. Radius of gyration (*R*_g_) and solvent accessible surface area (SASA) were calculated using tools within the GROMACS simulation package. Visualization was performed using PyMOL (the PyMOL Molecular Graphics System, Schrödinger, LLC).

## Results

### Ancestral Sequence Reconstruction

**Figure 1:**
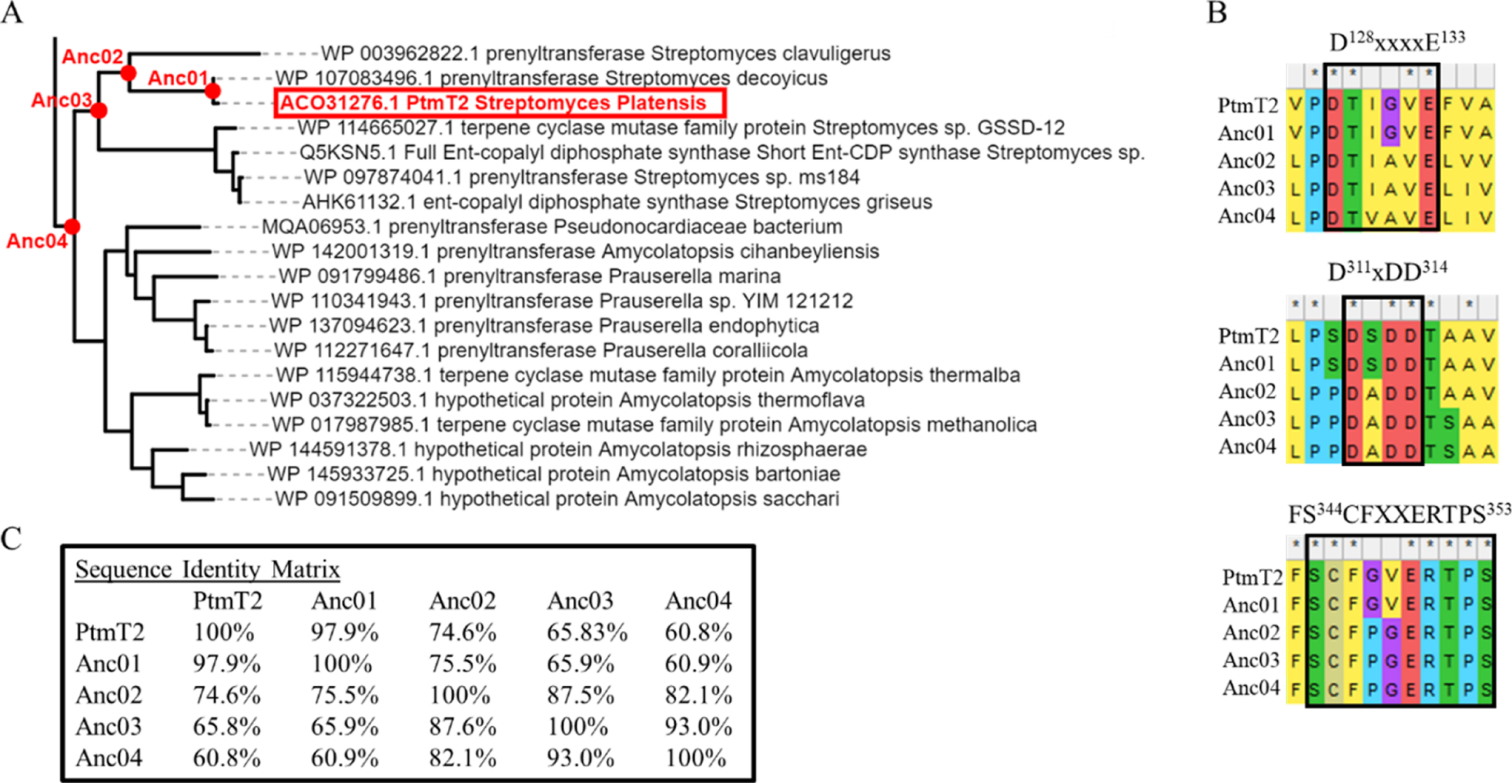
(A) Partial phylogenetic tree of PtmT2 (template sequence in the red box, full tree given in Supporting Information, Figure S1). The reconstructed ancestral sequences are indicated in red and labeled on their corresponding nodes. (B) Alignment of extant and ancestral protein sequences, showing key motifs: metal binding residues (D^128^xxxxE^133^), catalytic residues (D^311^xDD^314^), and loop (S^344^CFxxERTPS^353^) capping the active site. Complete alignment is shown in Supporting Information, Figure S2. (C) Sequence identity matrix of PtmT2 with the reconstructed ancestors.

**Figure 2:**
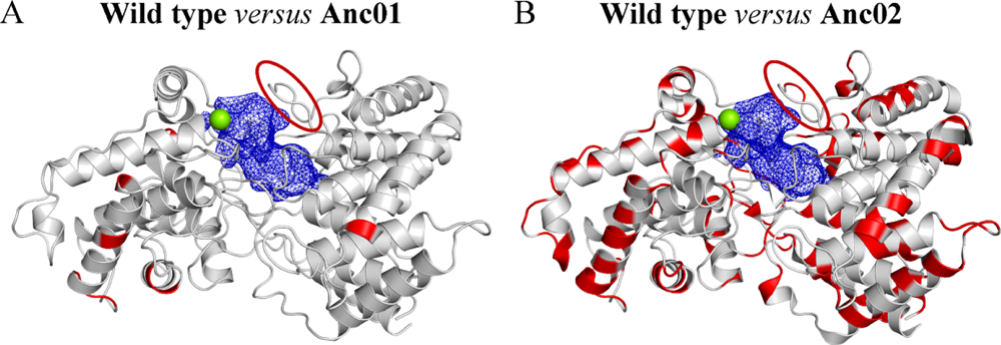
Positions of amino acid alterations (red) mapped onto the wild-type structure (gray cartoon) for Anc01 (panel A) and Anc02 (panel B). The manually docked Mg^2+^ ion (see below for details) is represented as a green sphere, whereas the active site cavity is depicted as a blue color mesh. The loop (residues 344–353) capping the active site is encircled in red.

The amino acid sequence of PtmT2 (GenBank: A0A023VSF1) from *S. platensis* was used as a template to create a multiple sequence alignment and phylogenetic tree. The search of homologous enzymes with a high sequence identity to PtmT2 yielded few hits. A mere five sequences had a sequence identity with PtmT2 of 50% or higher, highlighting the fact that bacterial diterpene cyclases remain underexplored.^42^ Therefore, the final alignment generated with the MUSCLE algorithm in MEGA-X^24,25^ consisted of 35 sequences with a typical sequence identity between 40 and 50%. The corresponding phylogenetic tree is shown in Supporting Information, Figure S1. Despite many of the sequences being labeled prenyltransferases, upon analysis, the sequences in the bottom half of the phylogenetic tree (shown in Figure 1A) contained the characteristic class II terpene cyclase motif (DxDD, Figure 1B). From the tree, four upstream nodes were selected corresponding to putative ancestral enzymes (referred to as Anc01-04). The sequence identity of these ancestral variants to extant PtmT2 is 97.9% (Anc01), 74.6% (Anc02), 65.8% (Anc03), and 60.8% (Anc04), respectively (Figure 1C). Spatial distribution of mutations is shown in Figure 2 for Anc01 and Anc02, that were functional and expressible (*vide infra*).

For the ancestral sequences, the D^311^xDD^314^ and D^128^xxxxE^133^ catalytic motifs (Figure 1B) described earlier,^20^ as well as the interior of the active site were confirmed to have remained essentially preserved during the reconstruction process (see Figure 2A,B for Anc01 and Anc02, respectively). For the D^311^xDD^314^ motif, Ser at position 312 in wild type and Anc01 was changed to an Ala in Anc02, Anc03 and Anc04.

**Figure 3:**
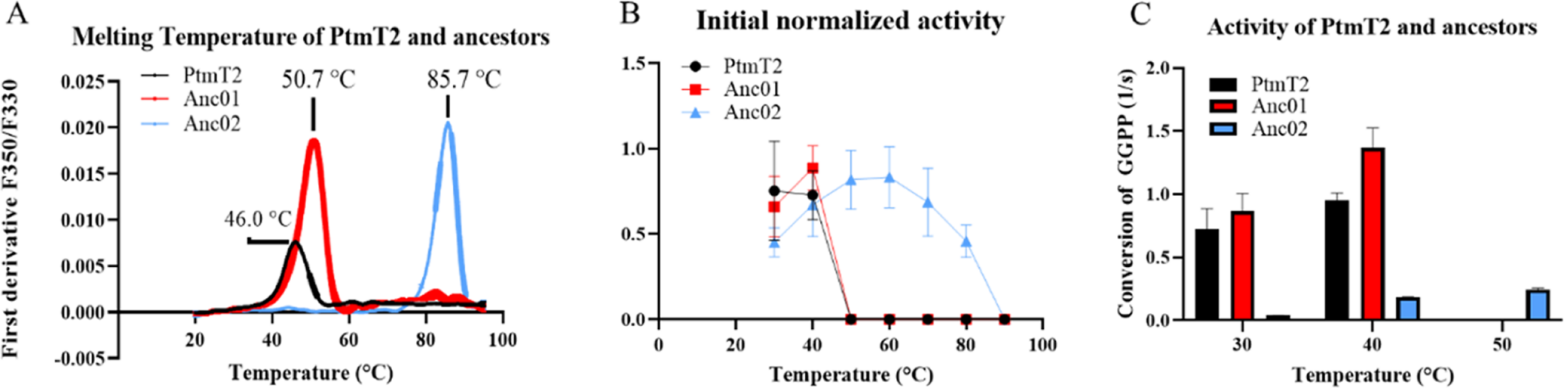
Thermostability and activity of ancestral diterpene cyclases compared to modern enzymes. (A) Thermal unfolding curves (technical triplicate) of PtmT2, Anc01, and Anc02. F350/F350 refers to the fluorescence readout at these wavelengths. (B) Normalized kinetic data given to each enzyme’s maximum conversion rate (*N* = 3). (C) Apparent initial absolute rates (*N* = 3) given as substrate conversion per second for PtmT2, Anc01, and Anc02 at the given temperatures. For clarity, data are shown for 30 to 50 °C (at higher temperatures, PtmT2 and Anc01 are inactive).

The expression of the putative ancestral sequences was performed in *E. coli* after which purification was performed using His_6_-tag and Ni-NTA beads (Supporting Information Figure S3A). The two oldest ancestors (Anc03 and Anc04) remained in the insoluble fraction during protein expression and were not able to be purified (Supporting Information Figure S3B). Therefore, further experiments were done with Anc01 and Anc02. The protein yield of all three proteins was similar after purification, approximately around 15 mg/mL. To analyze protein stability, we performed nanoDSF experiments monitoring the change in tryptophan fluorescence at 330 and 350 nm upon unfolding of the protein (Figure 3A). The ancestral proteins showed enhanced thermostability: Anc02 had a large change in the melting temperature (Δ*T*_m_) of 39.7 °C, with a *T*_m_ of 85.7 ± 0.0 °C, compared to 46.0 ± 0.1 °C displayed by extant PtmT2. On the other hand, Anc01 differing to the extant enzyme with a mere seven mutations had an Δ*T*_m_ of 4.9 ± 0.2 °C (*T*_m_ of 50.7 ± 0.2 °C, Figure 3A).

To investigate whether the ancestors’ increased thermostability was associated with a change in activity as a function of temperature, kinetic experiments were performed analyzing conversion of the GGPP substrate into product *ent*-CPP. To determine the optimal reaction temperature, enzymatic reactions were performed from 30 to 90 °C, with increments of 10 °C (Figure 3B). PtmT2 has the highest activity around 40 °C and Anc01 has a similar activity as the wild type at 40 °C (Figure 3B). Anc01 and PtmT2 displayed no activity at 50 °C, whereas Anc02 with its higher stability reaches its optimal normalized activity at this temperature (approximately 50–60 °C). Remarkably, ancestral PtmT2 even showed a residual activity up till 80 °C (Figure 3B). In terms of absolute activities, PtmT2 and Anc01 displayed similar reaction rates, whereas Anc02 was considerably slower with a 9- to 11-fold reduction in rate at 30 °C (Figure 3C). At 40 °C, Anc02 showed a 5-fold reduction in rate, but retained activity throughout a large range of temperatures, with a shift in the optimal temperature. The activity *versus* temperature profile of the ancestors is consistent with the measured thermostability of the proteins (*T*_m_ of 46.0 °C for wild type and 50.7 and 85.7 °C for the two ancestors, respectively). We reasoned that PtmT2, Anc01, and Anc02 could serve as a model system to understand molecular determinants contributing to thermoadaptation, which would require structural information.

### Homology Modeling and Metal-Binding Site Identification

The three-dimensional structures of the two functional and expressible ancestors (Anc01 and Anc02) were computationally modeled based on the template X-ray crystal structure of PtmT2 (PDB ID: 5BP8(20)). Modeled three-dimensional structures of Anc01 and Anc02 were found to be largely similar compared to the wild type’s experimentally determined structure (Supporting Information, Figure S4), with RMSD differences of 0.4 and 0.7 Å, respectively. The quality evaluation of the models was done with the inbuilt *Z*-scoring function in YASARA. The evaluation scores (Supporting Information, Table S4) collectively suggest that the models have good quality and are suitable to use for further computational analysis.

**Figure 4:**
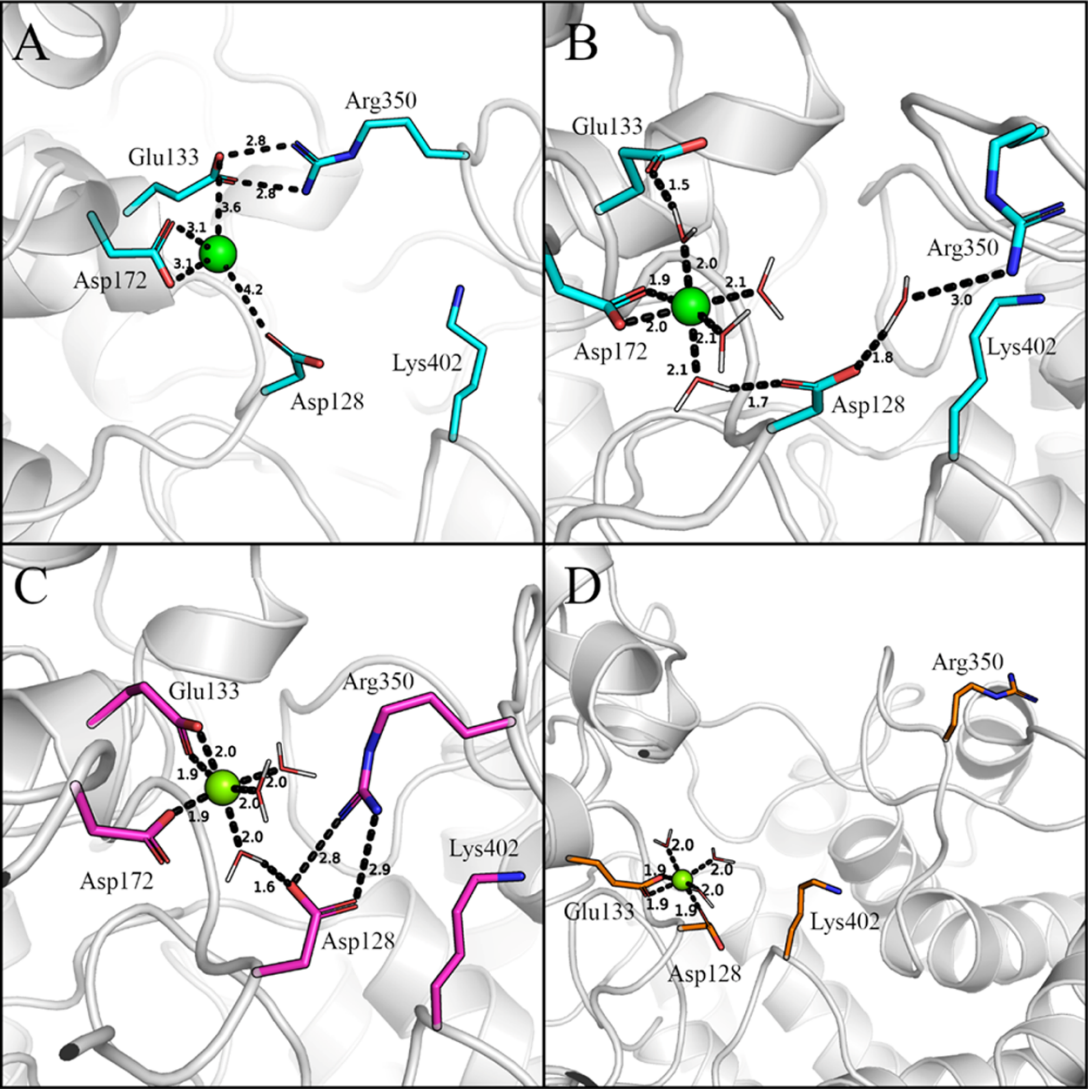
Manually docked Mg^2+^ ion-binding pose in PtmT2 before (panel A) and after (panels B, C, and D for PtmT2, Anc01, and Anc02, respectively) 10 ns MD simulations. Key residues are represented in sticks and metal ions as green spheres. Water molecules are represented in lines. Distances are given in Å. In Anc02 (panel D), Asp172 is mutated to Gly (not shown).

The catalytic metal is not visible in the crystal structure of PtmT2. Other experimental approaches for the identification of a Mg^2+^ metal-binding site in PtmT2 have been proven to be challenging: a few potential binding sites were proposed based on computational analysis of the structural differences between type I and II diterpene cyclases.^20^ Possible binding sites of Mg^2+^ ion were explored herein by a combination of metal ion-binding (MIB) web server^33,34^ and extensive docking together with MD simulations. Using the fragment transformation method inbuilt within MIB, a total of 10 binding sites were predicted for a Mg^2+^ ion. Most of the high-scoring sites reside on the protein surface, without any structure–activity relationship with respect to the proposed catalytic mechanism.^20^ In fact, none of the predicted binding poses (Figure S5, Supporting Information) show any structure–activity relationship, allowing us to discard all of the metal ion-binding poses predicted by the MIB web server. Previously D^128^xxxxE^133^ was identified as a potential Mg^2+^ binding motif,^20^ thus we manually docked the Mg^2+^ ion between the residues Asp128, Glu133, and Asp172 in wild type. The manually docked metal-binding pose (Figure 4A) was subjected to a short 10 ns MD sampling run to check the structural stability of the bound Mg^2+^ ion.

After MD simulations, the metal ion preserved its initial coordination in the manually docked pose as shown in Figure 4B, albeit with interactions between the metal, Asp128, and Glu133 mediated by water molecules. Furthermore, Asp128 interacts with Arg350 situated on the loop (residues 344–353) capping the active site through another water molecule as shown in Figure 4B. The salt bridge between Glu133 and Arg350 was lost after 10 ns MD simulations, with the side chain of Glu133 slightly turned so that the oxygen atoms coordinate with the metal ion through a water molecule. This stable metal-binding pose is consistent with what has previously been anticipated.^20^ Following the procedure for wild type, the Mg^2+^ ion was docked in Anc01 and Anc02, and 10 ns MD simulations were performed to assess structural stability.

A similar binding pose was observed in Anc01 (Figure 4C), but Glu133 and Asp128 directly couples to the metal ion and Arg350 without any water molecules, respectively. Similarly, in Anc02, Asp128 and Glu133 are also directly interacting with the metal without the involvement of any water molecules (Figure 4D). Furthermore, the Arg350 on the loop moved away from the negatively charged metal-coordinating residues, leaving the active site entrance widened when compared to the wild type and Anc01.

### Analysis of Structural Rigidity by MD Simulation

To understand if the observed difference in the thermostability of wild type and ancestors (Figure 3A) is related to structural rigidity, a total of 18 MD simulations were performed for 1 microsecond length, with 3 replica simulations for each variant (PtmT2, Anc01, and Anc02). The wild type, Anc01, and Anc02 were active to 40 °C, according to experimental data. Wild type and Anc01 loose activity as the temperature increases, but Anc02 remains active at 70 °C. MD simulations were performed at 303 K (30 °C) and 343 K (70 °C) to determine regions with high conformational flexibility, which we hypothesized to affect the thermostability and in turn, activity. The structures were simulated with a bound metal ion and without a substrate (Materials and Methods). The obtained trajectories were used to calculate the RMSD, RMSF, *R*_g_, SASA, and number of hydrogen bonds.

**Figure 5:**
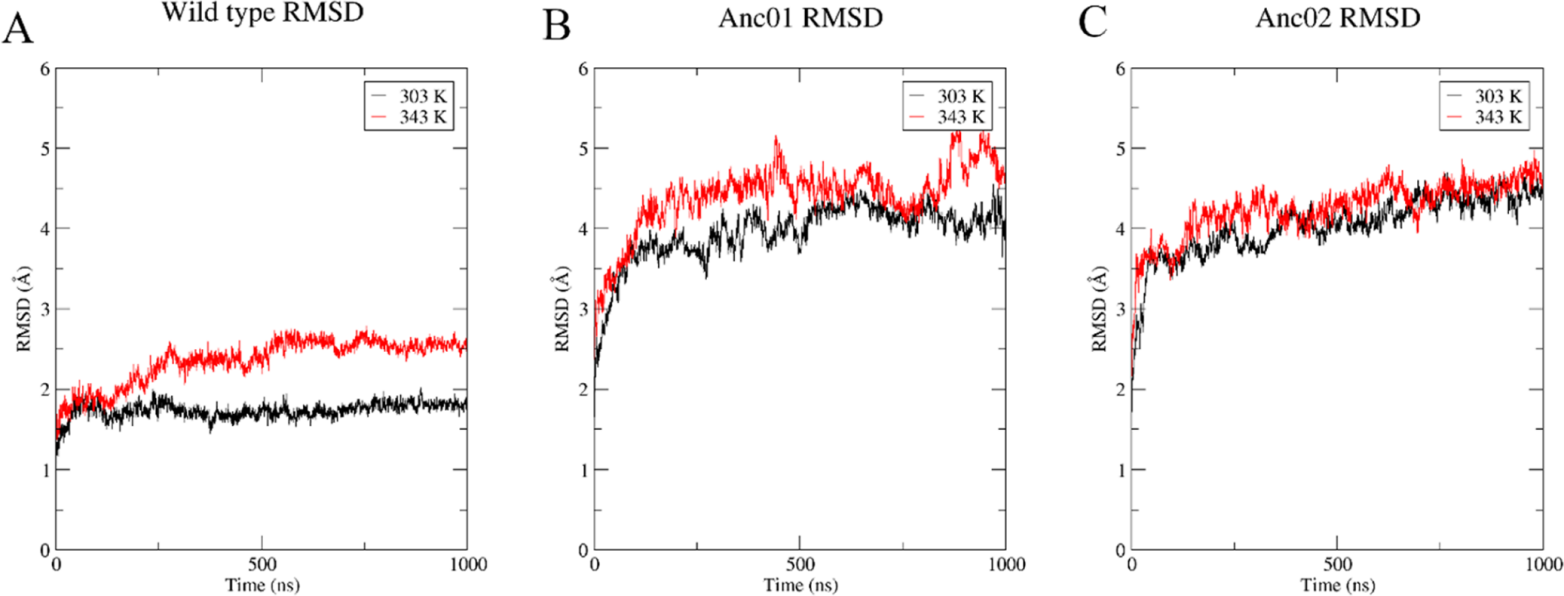
Average Cα RMSD compared to the respective starting structure is shown for wild type, Anc01, and Anc02 at different simulation temperatures in panels A–C, respectively. Supporting Information, Figure S6 contains plots of individual replicates.

The average RMSD values in the wild type increased from 1.7 ± 0.1 to 2.4 ± 0.2 Å with an increase of the temperature from 303 to 343 K (Figure 5A). Wild-type simulation shows a slight increase (to approx. 0.7 Å) in RMSD after ∼150 ns of simulation at 343 K. This increased RMSD implies that wild type accesses a different conformation at higher temperatures than at room temperature. With a temperature increase from 303 to 343 K, the average RMSD values for Anc01 increased slightly from 4.0 ± 0.3 to 4.4 ± 0.4 Å (see Figure 5B, homology models of ancestral enzymes were used as described in materials and methods). Furthermore, the MD simulations at both temperatures were not converged in Anc01, and this was particularly noticeable in the last 200 ns MD simulations. Notably, the RMSD difference between 303 and 343 K in Anc02 is 0.1 Å (4.1 ± 0.4 Å at 303 K and 4.2 ± 0.3 Å at 343 K), indicating that this ancestor is relatively stable at high temperatures (Figure 5C).

Exposing nonpolar residues to water has a negative impact on structural stability and is critical for protein unfolding. The contact area between biomolecules and the solvent is measured by the SASA, which thus may be used to determine structural integrity.^43^ We estimated the SASA at 303 and 343 K (Supporting Information, Table S5), and the difference between the variants (*i.e.,* ΔSASA_343–303K_) was small (260, 450, and 500 Å^2^ for wild type, Anc01, and Anc02, respectively). The *R*_g_, like the SASA, is a measure of protein structural compactness.^44^ The difference in the calculated *R*_g_ (Supporting Information, Table S5) between the variants at the two temperatures studied is likewise negligible (Δ*R*_g 343–303K_ of 0.1, 0.2, and 0.2 Å for wild type, Anc01, and Anc02, respectively), demonstrating that PtmT2 and variants are structurally conserved at both 303 and 343 K. The rigidity of the protein structure and the specificity of intramolecular interactions are both conferred *via* hydrogen bonding. Hydrogen bonds were estimated to assess structural stability and protein unfolding at 343 K. As shown in Supporting Information, Table S5, the difference in the number of hydrogen bonds between the two temperatures for wild type and variants is insignificant (ΔHydrogen-bonds_343–303K_ of −6 ± 8, −5 ± 7, and 0 ± 7 for wild type, Anc01, and Anc02, respectively) which further supports the hypothesis that these proteins were structurally stable in the simulations.

To determine any conformational differences between the variants, we next performed RMSF analysis of wild type and variants (Supporting Information, Figures S7 and S8). The RMSF is a weighted average of the displacement of a single atom or a group of atoms from the reference structure over time.^45,46^ RMSF calculations often require rigid-body alignment of the structures in each frame of the simulation to reference coordinates. The presence of subsets of the structure with substantial conformational changes, such as loops, makes the rigid-body alignment sensitive. High RMSFs might indicate that the whole structure fluctuates or might reflect only large displacements of a small structural subset within an overall rigid structure.^45^ About 40% of the residues in wild-type and variant structures are loops and turns, with many of them residing on the surface (see Figure 2). As expected, mobility of these regions is associated with relatively high RMSF values (see Supporting Information, Figures S7 and S8). Among the flexible regions, the loop containing the residues 344–353 caught our attention as it showed similar RMSF at 303 and 343 K for Anc02 (Supporting Information, Figure S8), in contrast to wild type and Anc01 (Supporting Information, Figure S7). Furthermore, as this loop (344–353) caps the active site, we hypothesized that it could be involved in thermoadaptation by affecting active site accessibility.

**Figure 6:**
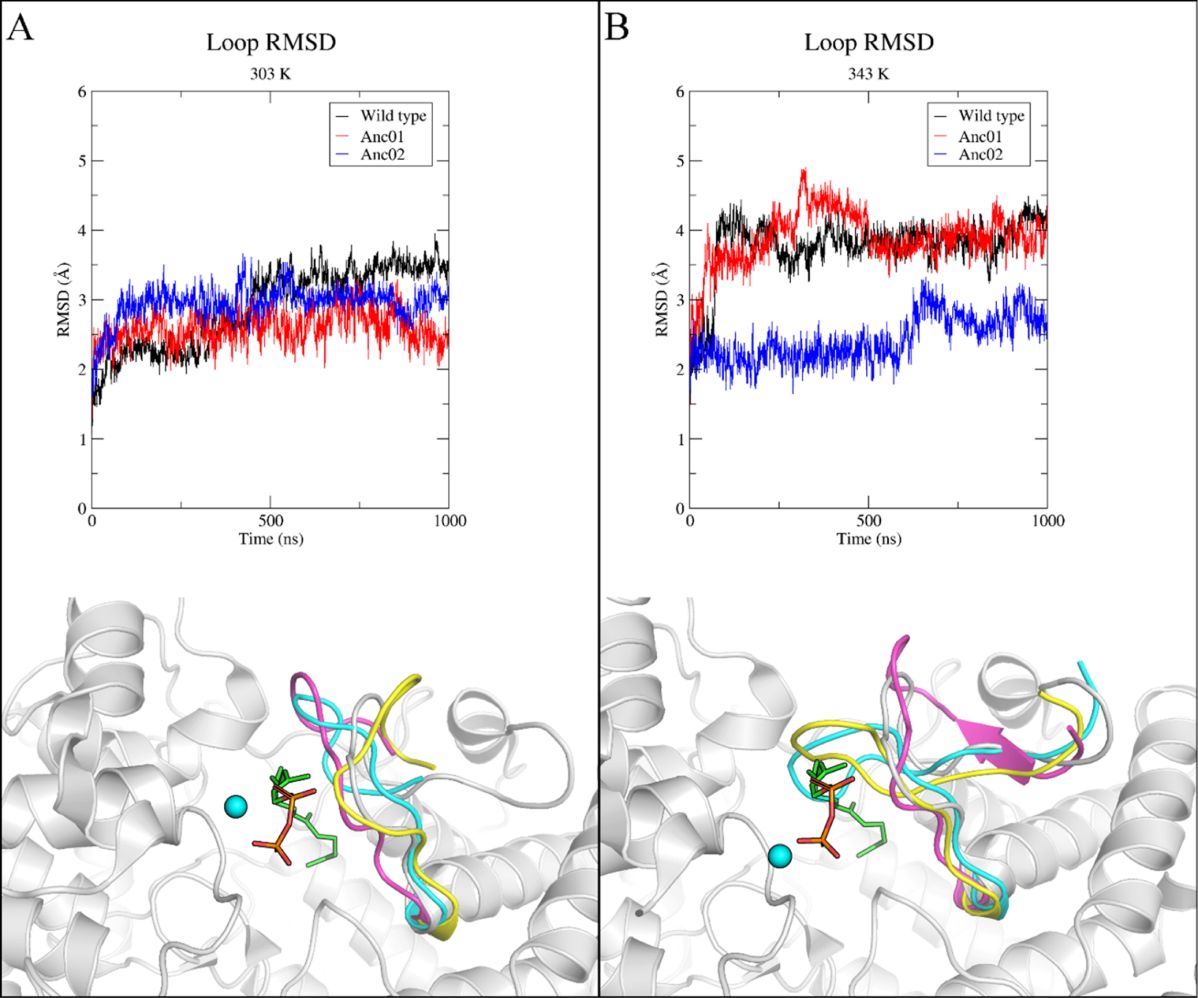
Backbone RMSD of loop at 30 °C (panel A, top) and 70 °C (panel B, top) together with representative loop conformations of 1 μs snapshots of PtmT2, Anc01, and Anc02 at 30 °C (panel A bottom) and at 70 °C (panel B bottom). X-ray crystal structure of PtmT2 (PDB ID: 5BP8) is shown in gray color for comparison. Loop conformations of residues 344–353 from 1 μs MD simulations in PtmT2 (cyan), Anc01 (yellow), and Anc02 (magenta) are shown. Metal ion is represented in spheres and the modeled GGPP substrate^20^ is shown in green sticks, with oxygen atoms in red and phosphorous in orange. The whole simulation trajectory (0–1000 ns) is shown in top graphs for clarity (triplicate, Supporting Information, Figure S9 contains plots of individual replicates).

To visualize the conformational heterogeneity of this loop (residues 344–353) that forms the “top” of the active site (Figure 2), representative three-dimensional structural snapshots from the 1 microsecond trajectories at 303 and 343 K were superimposed. For visualization, a bound substrate (GGPP) was generated using a binding mode previously reported.^20^ At 30 °C, the loop and metal ion-binding motif (Asp128, Glu133) remain similar in all variants compared to the X-ray crystal structure (see Figure 6A). In this confirmation, the loop resides in a productive capped conformation compatible with substrate binding and catalysis.^20^ Furthermore, all the key interactions to the metal ion described above (see Figure 4B) are retained. The loop backbone’s average RMSD was found to be the same (2.9 ± 0.6 Å for wild type, 2.6 ± 0.2 Å for Anc01, and 2.9 ± 0.3 Å for Anc02) in all variants at this temperature (30 °C), as shown in Figure 6A.

At 70 °C, this optimal complementarity between the loop (residues 344–353) and substrate was in stark contrast for wild type (average backbone RMSD 3.8 ± 0.4 Å) and Anc01 (average backbone RMSD 3.9 ± 0.4 Å), as shown in Figure 6B. In fact, after 100 ns of MD simulations at 70 °C for wild type and Anc01, the elevated backbone RMSD was found to be associated with a collapse of the loop into the active site (see Figure 6B). Conversely, the loop maintained in a productive conformation in Anc02 and was even observed to open further after a 600 ns MD simulation. In the collapsed loop conformation at an elevated temperature in wild type and in Anc01, the substrate may not be able to bind in the active site due to the steric clashes with the loop. However, in Anc02, the loop remains in a productive conformation allowing the substrate to bind in the active site for cyclization to occur. Thus, our results reinforced the hypothesis that the activity of these variants is, at least in part, due to the particular configuration of this loop.

**Figure 7:**
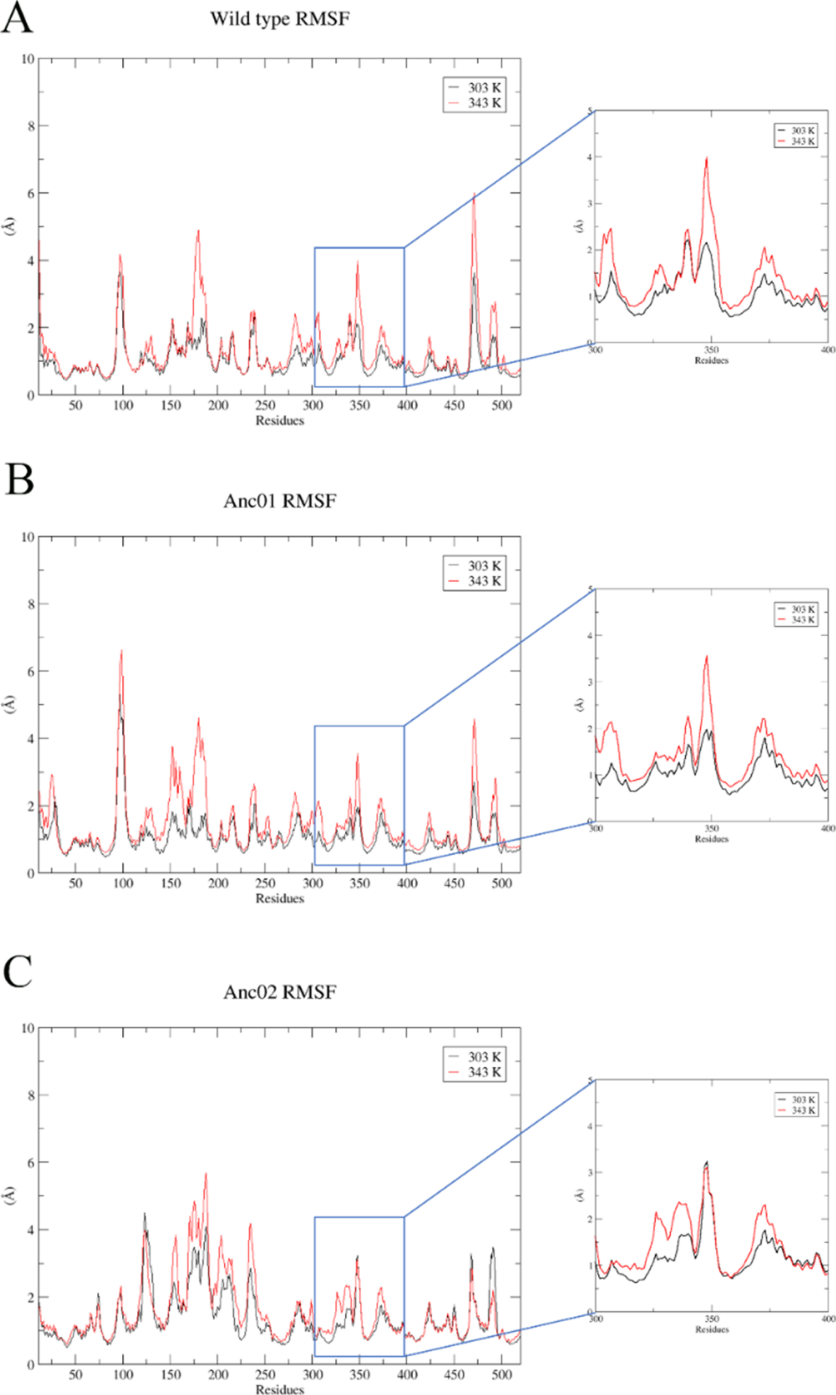
Average backbone RMSF of wild type (A), Anc01 (B), and Anc02 (C) at different simulation temperatures. Residues 300–400 for wild type, Anc01, and Anc02 are shown in enlarged sections of the corresponding panels.

Among the loop residues, Val^348^ (wild type and Anc01) and Gly^348^ (Anc02) displayed the largest RMSF values. Val^348^ fluctuates not more than 2.15 ± 0.1 and 1.97 ± 0.3 Å at 303 K, but 3.98 ± 0.6 and 3.55 ± 1.2 Å at 343 K in wild type and Anc01, respectively, as shown in Figure 7A,B (for clarity, we have highlighted residues 300–400). However, RMSF of Gly^348^ in Anc02 as 3.23 ± 1.0 and 3.10 ± 0.4 Å at 303 and 343 K, respectively, as shown in Figure 7C.

**Figure 8:**
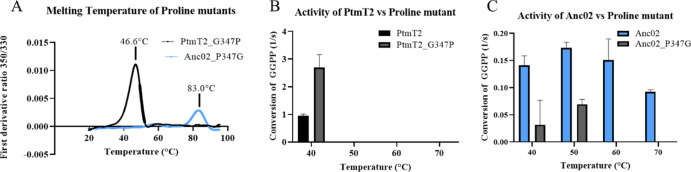
(A) Melting temperature of PtmT2_G347P and Anc02_P347G. (B) Activity of PtmT2 and PtmT2_G347P at 40 to 70 °C (*N* = 3). (C) Activity of Anc02 compared to Anc02_P347G at 40 to 70 °C (*N* = 3).

A difference in the loop between PtmT2, Anc01, and Anc02 is at the G^347^V^348^ positions: in Anc02, these residues are mutated to a Pro and Gly, respectively (Figure 1B). We hypothesized that the proline substitution in Anc02 provided a rigidifying effect to the loop in preventing its collapse at high temperatures. To test this hypothesis, we mutated the proline in Anc02 back to the wild-type glycine residue, creating the Anc02_P347G variant. Similarly, we added the proline mutation in wild-type PtmT2, creating the PtmT2_G347P variant. The impact of the Pro/Gly substitutions on the melting temperature was found to be small: the *T*_m_ of the PtmT2_G347P mutant increased with 0.6 °C compared to the extant protein, whereas the switch of the proline for a glycine in Anc02 led to a modest 2.7 °C decrease in *T*_m_ (Figure 8A). The effect of these mutations on activity were tested from 40 to 70 °C with a 10 °C increment (Figure 8B,C). Interestingly, the loop mutant PtmT2_G347P showed a 2.7-fold increase in activity compared to wild-type PtmT2 (Figure 8B). At 40 °C, Anc02 had a higher activity than Anc02_P347G (Figure 8C). The activity increased in both the Anc02 and its P347G variant at 50 °C, a temperature at which the extant enzyme and Anc01 display no activity (Figure 3B,C). Interestingly, the activity in the loop mutant of ancestral PtmT2 completely disappeared at 60 and 70 °C (Figure 8C).

## Discussion

Enzymes are at the heart of sustainable development in enabling industrial manufacturing of chemicals, biofuels, and material precursors under mild reaction conditions and with minimal generation of waste and hazardousness in accordance to the twelve principles of green chemistry.^47−49^ Terpenes are receiving considerable attention as renewable hydrocarbons with applications as flavors, fragrances, green chemical building blocks, and as antibiotics and antiviral compounds.^49,50^ Plants have long been cherished for their natural oils enriched in (multi)cyclic terpenes and terpenoids with potent biological activities; and more recently, genome mining has started to unravel the great diversity of bacterial terpene biosynthetic machineries with important implications for industrial biotechnology.^51^ A prominent example is the diterpene cyclase PtmT2 from *S. platensis* involved in the biosynthesis of the antibiotics platensimycin and platencin.^20,52^ A crystal structure of PtmT2 has recently been reported by Rudolf *et al.*(20) benefiting the understanding of the molecular mechanism of catalysis, crucial for further enzyme engineering process to create more efficient PtmT2 enzyme variants.

Enzyme engineering^53^ is the key to accelerate slow terpene biosynthesis and to create more stable enzymes^54^ opening up opportunities in synthetic biology.^55−57^ Previously, directed evolution followed by a screening campaign led to a terpene synthase with a 12 °C increase in the melting temperature (*T*_m_).^54^ As an orthogonal track to directed evolution, we used ancestral sequence reconstruction as an enzyme engineering tool to enhance *T*_m_ of the bacterial diterpene cyclase PtmT2, as evolvable ancestral biocatalysts are expected to be robust in order to tolerate additional mutations.^8^ Using this approach, we generated a hyperstable ancestral diterpene cyclase with a remarkable 40 °C increase in the melting temperature, by generating and analyzing just four sequences of putative ancestors to PtmT2.

In contrast to a pure consensus approach,^11^ ancestral sequence reconstruction can generate the most probable sequence that gave rise to the modern proteins observed today. We previously showed that the consensus sequence of a family of class I terpene cyclases was catalytically inactive,^58^ in contrast to ancestral enzymes which showed higher *k*_cat_/*K*_M_ compared to modern biocatalysts. Ancestral sequence reconstruction is heavily reliant on the number of homologous sequences found in databases. The BLAST search of PtmT2 yielded very few hits of high similarity, stressing the fact that bacterial diterpene cyclases remain underexplored.^51^ In fact, most of the proteins related to PtmT2 found were classified as prenyltransferases, although—at the moment of creating the phylogenetic tree—most of them were not experimentally studied. Upon analysis of the sequence alignment, it became apparent that at least half of the predicted prenyltransferases contained the DxDD motif which is distinctive for class II terpene cyclases.^49^ The phylogenetic tree generated in this work yielded four putative ancestral sequences, all of which retained the amino acids in the active site identified as significant by Rudolf *et al.*(20) including the DxDD and DxxxxE motifs. Out of the four investigated putative ancestral sequences, we were not able to express and purify the two oldest ancestors. We cannot exclude contributions from uncertainty stemming from sequence annotation (*i.e.*, prenyltransferase *vs* terpene cyclase) and its implication on tree building. Still, it is known that terpene cyclases can have both prenyltransferase and cyclase activity.^59^

The two functional ancestral enzymes (Anc01 and Anc02) were experimentally analyzed following expression in *E. coli* and subjected to *in silico* studies to shed light on possible molecular mechanisms contributing to thermoadaptation. As the location of the metal ion was not visible in the X-ray crystal structure (PDB: 5BP8),^20^ we were forced to analyze and confirm the metal-binding site prior to MD simulations. Metal ions play an important role in many cellular processes,^60,61^ for example, Na^+^, K^+^, Mg^2+^, and Ca^2+^ play structural^62^ roles, whereas transition metals such as Fe^2+^ and Cu^2+^ are often involved in both stabilization and catalysis.^63,64^ In the absence of a PtmT2 crystal structure with a bound metal ion, we conducted an exhaustive docking study to accurately identify the metal-binding site using the MIB web server. Unfortunately, the binding positions predicted by the MIB server did not yield any satisfactory binding poses which forced us to manually dock the Mg^2+^ ion adjacent to the D^128^xxxxE^133^ motif, which was previously identified as a potential Mg^2+^ binding site.^20^ We docked a Mg^2+^ ion at a distance of 3.1 Å to the oxygens of Asp172 and 3.6 and 4.2 Å to Glu133 and Asp128 oxygens, respectively. As shown in Figure 4A, the distances were chosen to generate a binding pose that could be used to emulate the previously proposed interactions.^20^ The metal ion reorients itself to coordinate to the Asp128, Glu133, and Asp172 (as described above) after a short 10 ns MD simulation on this binding pose. However, an interesting conformational change was observed for the loop residue Arg350, which was initially coordinated to Glu133 in the X-ray crystal structure. In the presence of a metal ion, Glu133–Arg350 interaction was lost during the MD simulation. This loss might be the result of Glu133 being attracted to a more positively charged metal ion. Despite this, Arg350 interacts with Asp128 through a water molecule, leaving the active site entrance open as shown in Figure 4B, which further explains the experimental mutagenesis data available for residue Asp128.^20^ Nonetheless, the Mg^2+^ ion was shown to have a stable octahedral coordination with two free water molecules, which may be substituted by oxyanions of the pyrophosphate unit of the substrate GGPP upon binding. These simulations allowed us to identify the potential metal-binding site, which was conserved within wild-type and ancestral variants of this protein and was consistent with the experimental mutagenesis data.^20^

None (Anc01, Figure 2A) or few (Anc02, Figure 2B) of the mutated residues between the ancestors and the extant protein were in the active site. In fact, the vast majority of mutations in the enzyme are present at the surface of the protein (Figure 2), whereas the helices pointing inward of the protein are mostly unchanged in Anc02. Therefore, the activity at higher temperature displayed by Anc01 and Anc02 is caused by mutations not directly assisting the carbocationic cyclization cascade. Nonetheless, the activity of Anc02 decreased 6-fold compared to the wild type raising the question of the impact mutations has on flexibility of the protein. We performed a temperature denaturing experiment to see if the difference in flexibility affected a protein’s ability to refold. The experimental data showed that none of the studied proteins were able to refold and all activity was lost after denaturing.

With a metal-binding site confirmed, we performed MD simulations at two different temperatures (303 and 343 K), aiming to understand possible molecular determinants contributing to thermoadaptation in ancestral PtmT2. Starting from *apo* crystal structure and homology models of extant and ancestral proteins, respectively, we found that the difference between the average backbone RMSD at 303 and 343 K in the wild type (0.7 Å) is somewhat bigger than in ancestors during simulations (0.4 and 0.1 Å in Anc01 and Anc02, respectively). In Anc02, the difference is insignificant, indicating structural rigidity which could allow the protein to withstand elevated temperatures. Enzyme denaturation typically occurs on a time scale ranging from tens of microseconds to milliseconds.^65^ Simulating such occurrences in real time is not yet feasible in a reasonable amount of time^66^ and as a result, we used shorter 1 microsecond MD simulation which was sufficient to observe loop areas of significant conformational change. Specifically, closer inspection of flexible parts of the protein structure for modern enzymes and ancestral variants thereof led to the identification of a flexible loop (residues 344–353), that acts as a gate to the active site cavity. The spatial conformation of this loop is identical in all three variants at 303 K. Interestingly, this loop is collapsed into the active site in wild type and Anc01 at 343 K, preventing substrate binding (as seen in Figure 6), incompatible with the previously hypothesized binding and catalytic mechanisms.^20^ The RMSF analysis also revealed that at 303 and 343 K, wild type and Anc01 were accessing two distinct loop conformations. The increased RMSF values at 343 K correspond to the observed loop collapse in the active site cavity. The loop residues in Anc02, on the other hand, remain stable regardless of temperature, corroborating the hypothesis that the loop (residues 344–353) conformation plays a significant role in enzyme activity. The RMSF analysis revealed that wild type and Anc01 followed a similar trend at both 303 and 343 K, with the loop fluctuations being much larger at 343 K. However, in Anc02, such fluctuations due to high temperature were significantly less which indicates the structural stability of Anc02 at high temperatures.

The Mg^2+^ ion is shown to shift position in the wild-type structure concomitantly with loop closure. One possible explanation for the shift of Mg^2+^ and collapse of the loop (residues 344–353) is the presence of a glutamate at position 351. The loop collapses into the active site with the glutamate being in the range to interact with the Mg^2+^ ion. The Anc01 and the wild-type structures have identical sequences in this loop and thus show the same tendency in the simulations. Anc02, however, does have a proline mutation in the loop which could explain the stability of the loop in this variant, which we confirmed by mutagenesis followed by kinetic experiments. The results showed a significant shift in activity in Anc02_G347P with a loss of activity above 60 °C. The proline mutation, upon introduction in PtmT2, caused a 2.7-fold activity increase in PtmT2, verifying the importance of the loop and of the stabilizing proline mutation in this region.

## Conclusions

In summary, herein, we reconstructed an ancestral class II terpene cyclase from *S. platensis* with a large increase in thermostability at 40 °C. This increase in thermostability is higher than previously achieved for terpene cyclases^58,67^ and is at par with billion year old reconstructed thermostable adenylate kinases.^6^ The increased stability achieved herein not only shows the potency of ancestral sequence reconstruction as a design technique to create thermostable proteins but also highlights its caveats dictated by the evolutionary stability–activity tradeoff. In the case of PtmT2, the rigidified structure of the ancestral enzyme results in a decrease in maximal activity compared to the modern biocatalyst. The increased rigidity allows the enzyme to retain activity at high temperatures by keeping a key capping loop in a productive and open conformation ready for substrate binding and catalysis. The loop (residues 344–353), consisting of the GVER motif, appears to be important for the stability and function of the protein at higher temperatures, as MD simulations show a collapse of this loop into the active site, blocking substrate access for the modern enzyme and the youngest ancestral protein. We verified by mutagenesis that a proline loop residue appearing in the ancestral PtmT2 is the key in mediating activity at high temperatures, as upon restoring this key proline back to glycine found in PtmT2 led to abolished activity at an elevated temperature. Retained activity at 50 °C concomitant with the small 2.7 °C decrease in the melting temperature displayed by the loop variant shows that the ancestral background contributes to stabilizing the protein backbone. Our work cements the importance of loops in enzyme catalysis and highlights the potential of ancestral sequence reconstruction to generate starting points in enzyme design with an inherently higher thermostability compared to its extant counterpart. The robust ancestral fold can then be subjected to further mutations to enhance activity and identify critical amino acids for the catalytic activity.

## Supporting Information Available

Full phylogenetic tree and sequence alignment; SDS–PAGE; homology models; visualization of Mg^2+^ ion-binding sites in PtmT2; replicate RMSD and RMSF plots; information on spatial positioning of flexible loops; primers, plasmids and strains used; *Z*-scores of the homology models; and SASA, radius of gyration, and hydrogen bond analyses (PDF)
