# Supplementary material for: Thermoadaptation in an Ancestral Diterpene Cyclase by Altered Loop Stability
Source: J Phys Chem B. 2022 May 18;126(21):3809–21. doi: 10.1021/acs.jpcb.1c10605 (PMC9169049; doi:10.1021/acs.jpcb.1c10605)
Supplement: Supplementary file 1 — jp1c10605_si_001.pdf [file jp1c10605_si_001.pdf]

Thermoadaptation in an Ancestral Diterpene Cyclase by Altered Loop Stability

*David A. Hueting[1,2], Sudarsana R. Vanga[1,2] and Per-Olof Syrén[1,2]**

[1]School of Engineering Sciences in Chemistry, Biotechnology and Health, Science for Life Laboratory, KTH Royal Institute of Technology, 114 28 Stockholm, Sweden.

[2]School of Engineering Sciences in Chemistry, Biotechnology and Health, Department of Fibre and Polymer Technology, KTH Royal Institute of Technology, 114 28 Stockholm, Sweden.

SUPPORTING INFORMATION

Table of contents:

Figure S1	Phylogenetic tree of PtmT2 reconstructed in MEGA-X	2
Figure S2	Full alignment of PtmT2, Anc01, Anc02, Anc03 and Anc04	3
Figure S3	SDS-Page PtmT2 and Ancestors	4
Figure S4	Superimposed homology models of Anc01 (cyan) and Anc02	5
Figure S5	Mg^{2+} ion binding sites in PtmT2	6
Figure S6	Replicate RMSD plots at 303 K and 343 K for wild type, and variants	7
Figure S7	Position of flexible loops and corresponding RMSF data for PtmT2 and Anc01	8
Figure S8	Position of flexible loops and corresponding RMSF data for Anc02	9
Figure S9	Replicate RMSD plots of loop at 303 K and 343 K for wild type and variants	10
Figure S10	Replicate RMSF plots at 303 K and 343 K for wild type and variants	11
Table S1	Primers used in this study	12
Table S2	Strains used in this study	12
Table S3	Plasmids used in this study	12
Table S4	*Z*-scores of the homology models of the ancestors	13
Table S5	Calculation of SASA and Radius of gyration	14

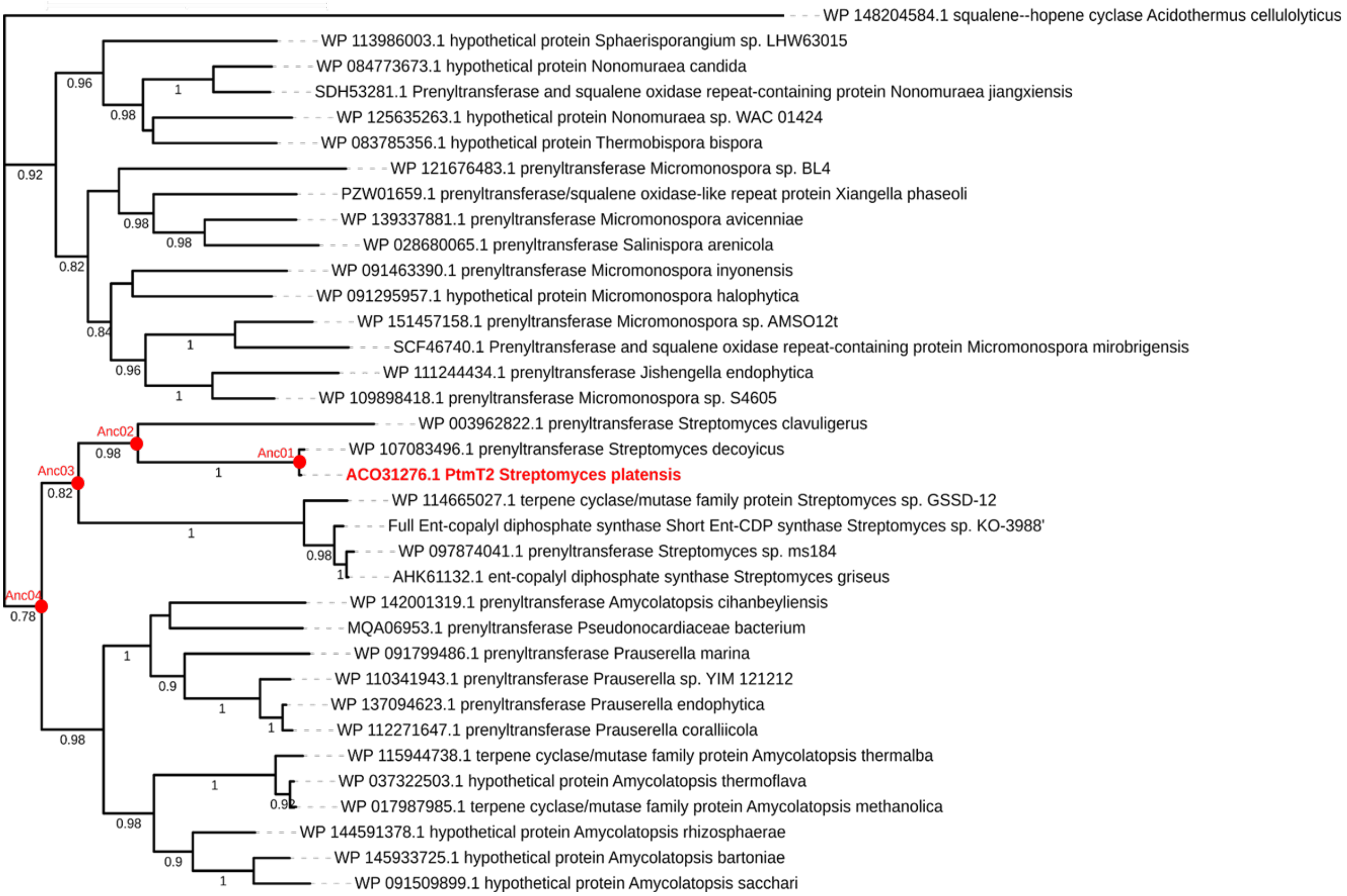

Figure S1. Phylogenetic tree of PtmT2 constructed in MEGA-X. The nodes of interest are annotated with Anc01, Anc02, Anc03 and Anc04 respectively. The values shown are bootstrap values (n=1000).

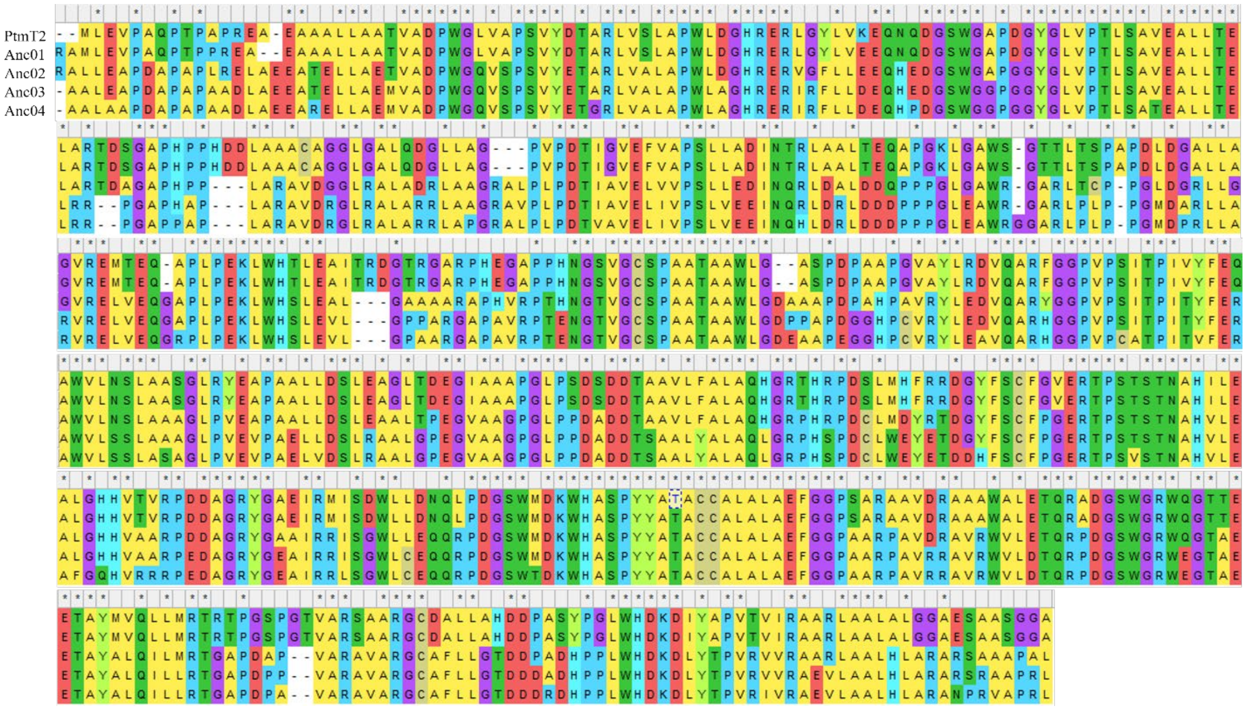

Figure S2. Full alignment of PtmT2, Anc01, Anc02, Anc03 and Anc04.

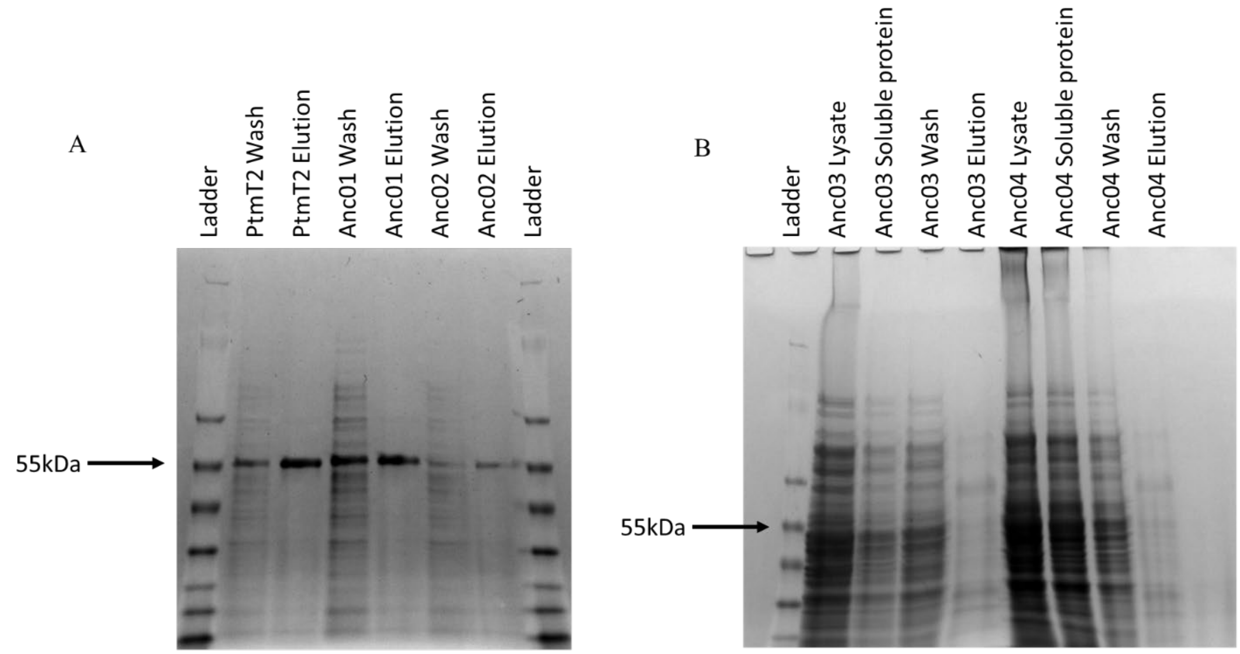

Figure S3. A SDS-Page gel of PtmT2, Anc01 and Anc02, showing wash fraction and elution fraction after Ni-NTA bead purification. Molecular weight of PtmT2 is 55.5 kDa. **B** SDS-Page gel of the purification steps of A3 and A4. The lysate, soluble fraction, wash and elution steps are shown. Ladder was SeeBlue Plus2.

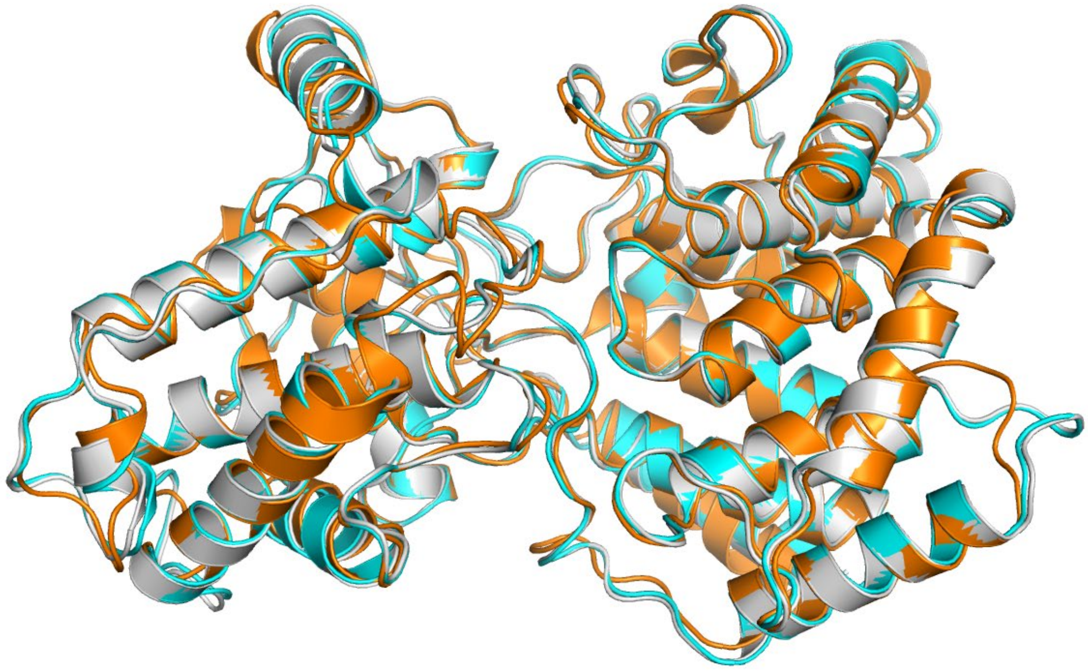

Figure S4. Superimposed homology models of Anc01 (cyan) and Anc02 (orange) onto the X-ray crystal structure (PDB ID: 5BP8) of PtmT2 (grey). The RMSD difference between wild type and Anc01 and Anc02 was calculated to be 0.4Å and 0.7Å, respectively.

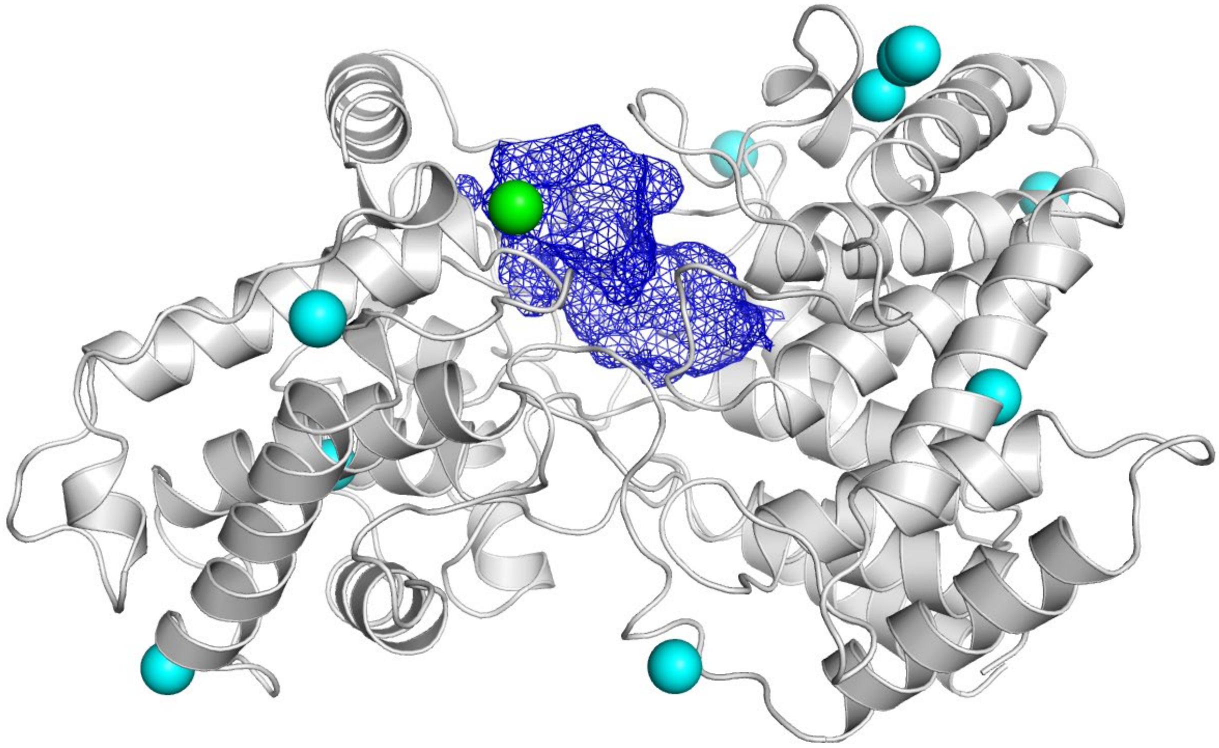

Figure S5. Mg^{2+} ion binding sites in PtmT2. The Mg^{2+} ion binding sites predicted by the Metal Ion Binding Site (MIB) server are shown by cyan color spheres. The manually docked Mg^{2+} is shown by the green color sphere. The active site location is depicted as a blue mesh.

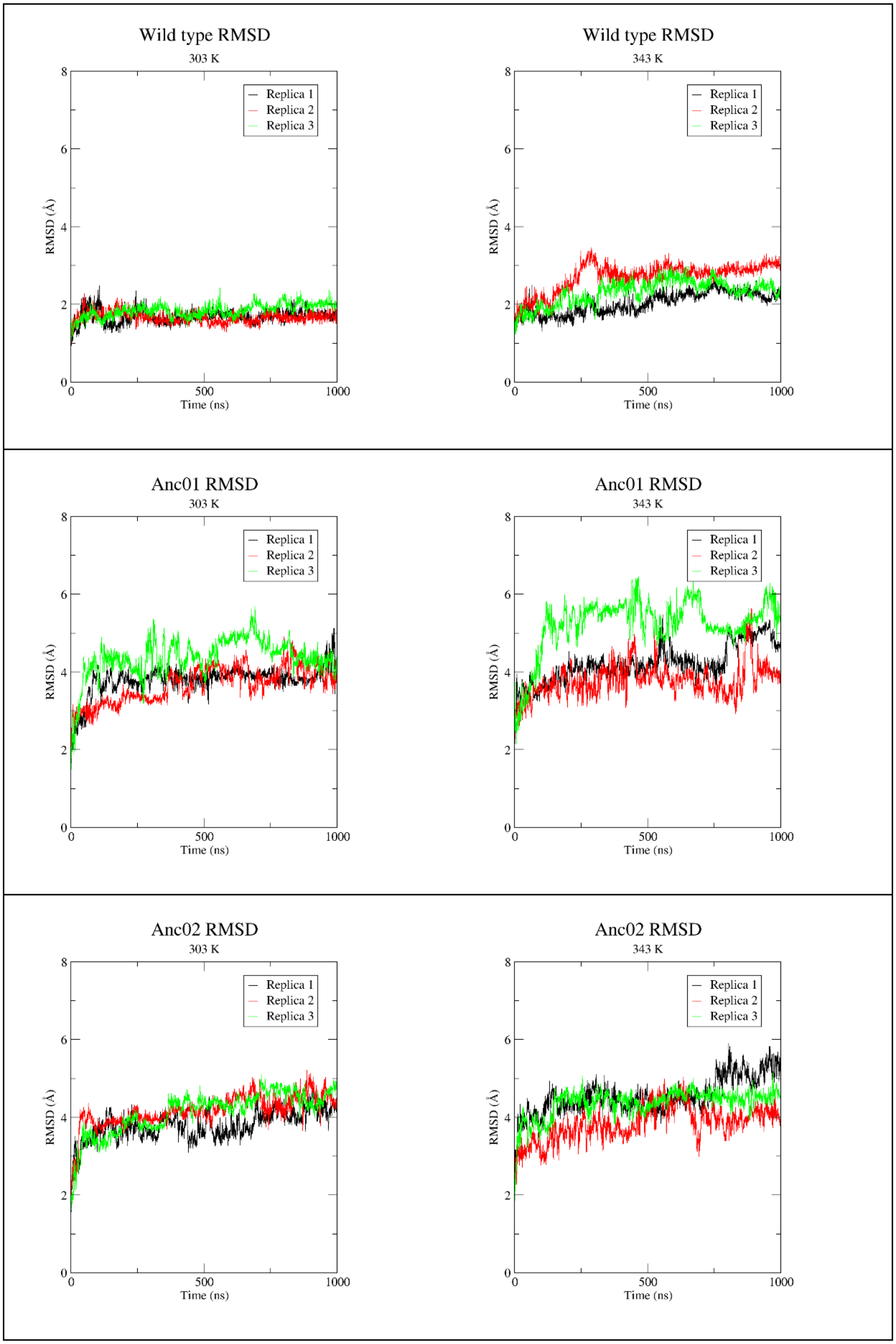

Figure S6. Individual replicate RMSD plots at 303 K and 343 K for wild type (*top*), Anc01 (*middle*) and Anc02 (*bottom*).

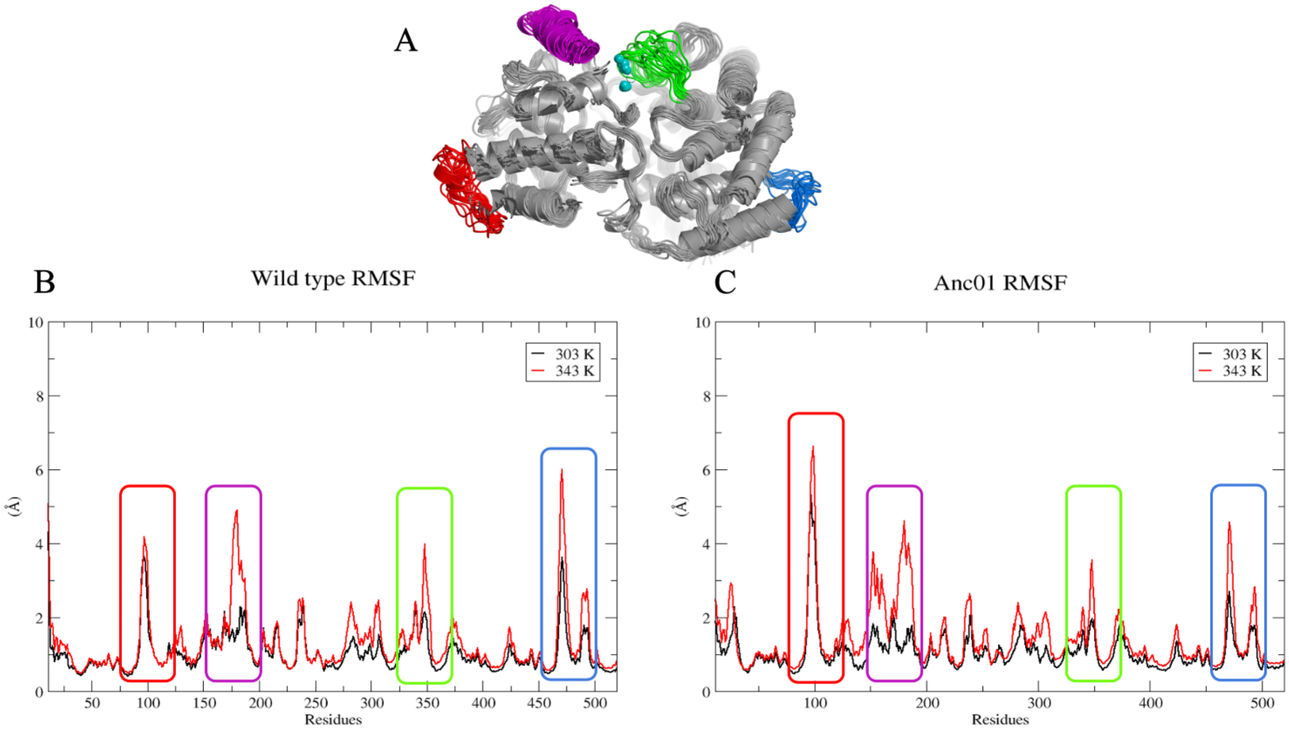

Figure S7. A total of 100 snapshots were superimposed from wild-type MD simulations (panel A). The flexible regions are colored, whilst the highly aligned helices are depicted in a grey cartoon and the metal ions are represented as cyan colored spheres. The color-coded loops in (A) correspond to RMSF data in wild type (panel B) and Anc01 (panel C). The loop capping the active site is shown in green.

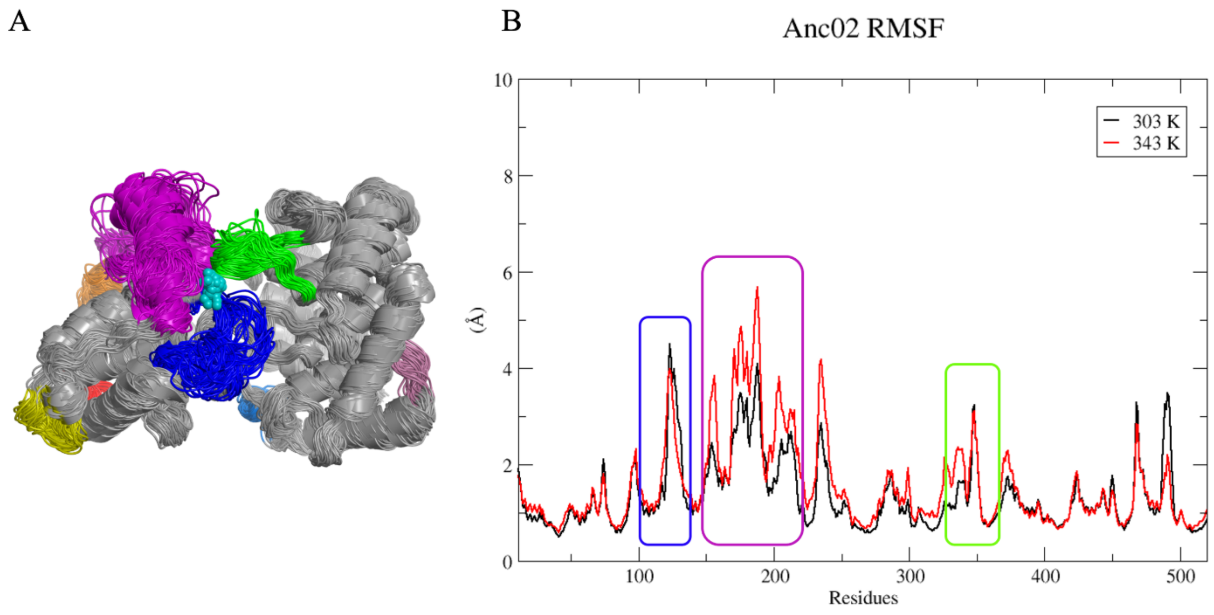

Figure S8. A total of 100 snapshots were superimposed from Anc02 MD simulations (panel A). The flexible regions are colored, whilst the highly aligned helices are depicted in a grey cartoon and the metal ions are represented as cyan colored spheres. The color-coded loops in (A) correspond to RMSF data in Anc02 (panel B). The loop capping the active site is shown in green.

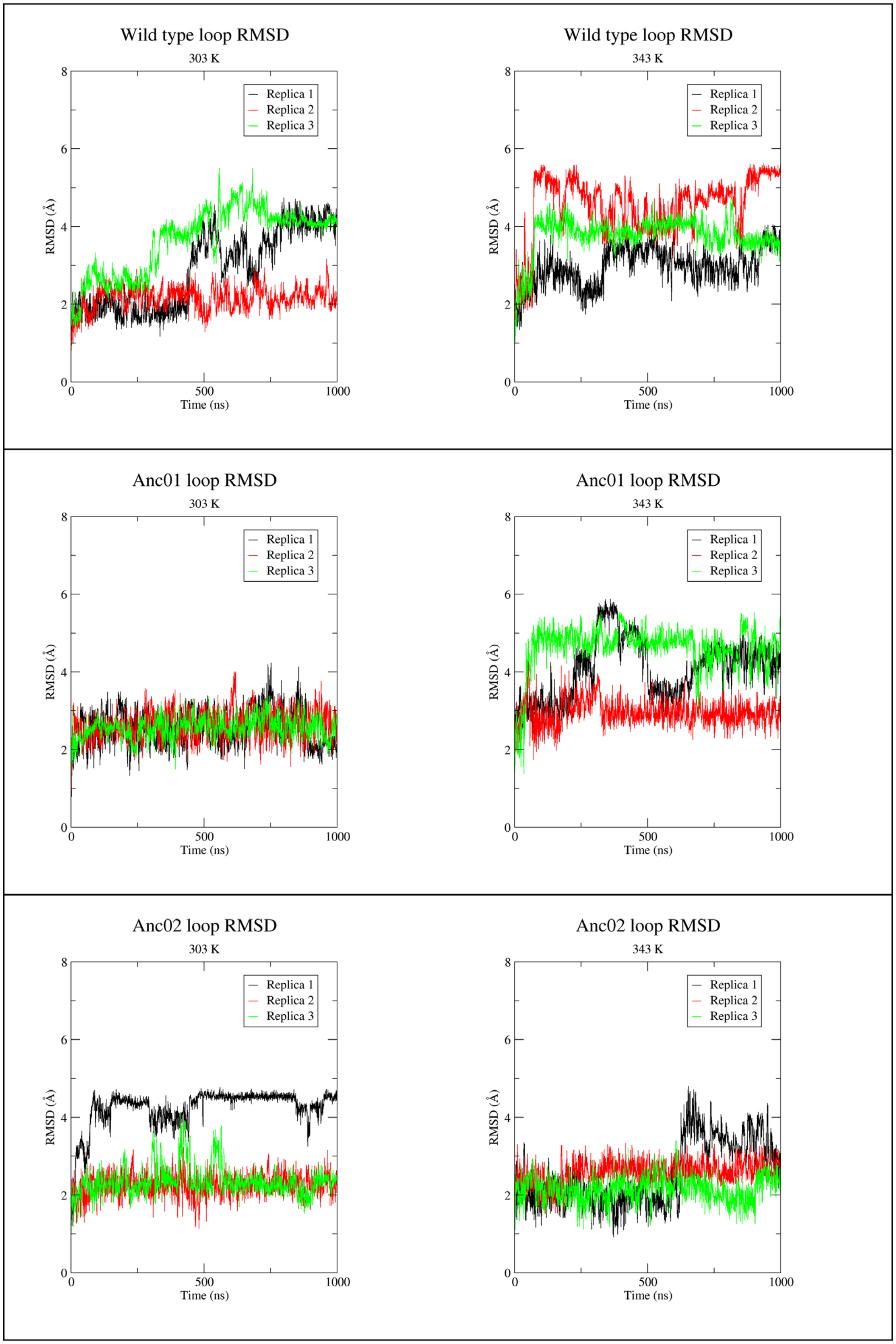

Figure S9. Individual replicate RMSD plots of loop at 303 K and 343 K for wild type (*top*), Anc01 (*middle*) and Anc02 (*bottom*).

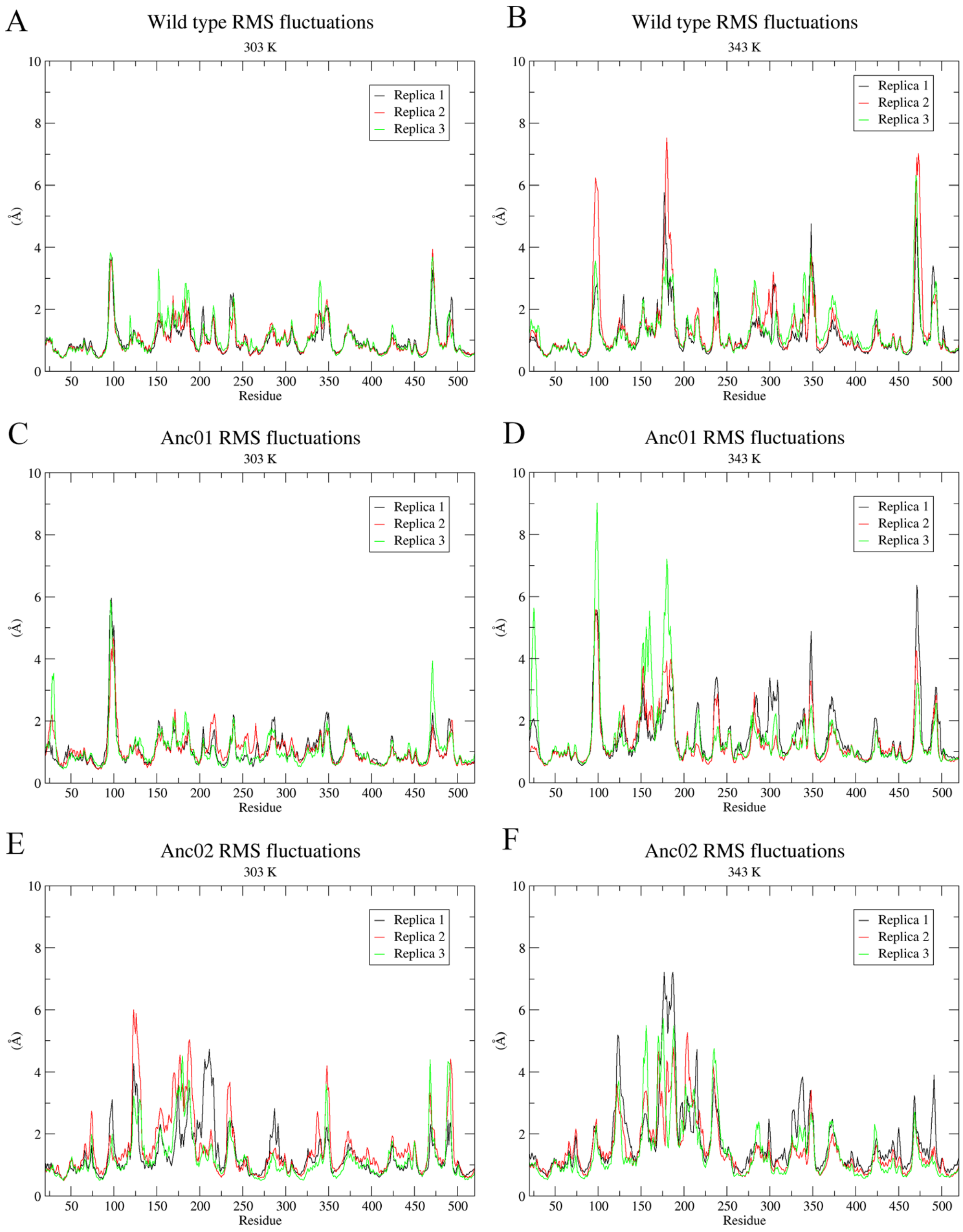

Figure S10. Individual replicate RMSF plots at 303 K and 343 K for wild type (*top; A and B*), Anc01 (*middle; C and D*) and Anc02 (*bottom; E and F*) respectively.

Table S1. Primers used in this study

Name	Sequence	Purpose
PtmT2_Proline_FW_Q5	TAGCTGCTTTCCTGTGGAACGTACC	Mutagenesis(G347P)
PtmT2_Proline_Rev_Q5	AAATAACCATCACGACGAAAATG	Mutagenesis (G347P)
Anc02_Proline_FW_Q5	TAGCTGTTTTGGGGGTGAACGTAC	Mutagenesis (P347G)
Anc02_Proline_Rev_Q5	AAATAACCATCGGTGCGATAATC	Mutagenesis (P347G)
T7Rev	CCTATATCGCCGACATCACC	Sequencing
PBRevF	GGTGATGTCGGCGATATAGG	Sequencing

Table S2. Strains used in this study

Strain	Purpose	Source
E. Coli XL1-Blue	Cloning and plasmid expression	Agilent
E. Coli C43(DE3)	Protein purification	Sigma Aldrich

Table S3. Plasmids used in this study

Name	Source
pET22b(+)_PtmT2	This study
pET22b(+)_Anc01	This study
pET22b(+)_Anc02	This study
pET22b(+)_Anc03	This study
pET22b(+)_Anc04	This study
pET22b(+)_Anc02_Pro	This study
pET22b(+)_PtmT2_Pro	This study

Table S4. *Z*-scores of the homology models of the ancestors. Individual components of the scoring function are shown.

	***Z*-Score**	
	**Anc01**	**Anc02**
Dihedrals	0.735	0.016
Packing 1D	-0.247	-0.411
Packing 3D	0.331	0.023
**Overall**	0.164	-0.147

Table S5. Calculation of SASA and Radius of gyration and hydrogen bonds for wild type and two ancestors over 1 microsecond MD simulations. Errors are standard deviations of corresponding replica simulations. 3 replica simulations were performed for each variant. Hydrogen bonds and the corresponding errors are rounded off to the nearest integer.

	SASA ($Å^2$)		Radius of gyration (*R*g) (Å)		Hydrogen bonds	
	30 ^{0}C	70 ^{0}C	30 ^{0}C	70 ^{0}C	30 ^{0}C	70 ^{0}C
Wild type	20729.0 ± 40	20471.0± 110	23.5 ± 0.0	23.4 ± 0.0	400 ± 4	394 ± 4
Anc01	21839.0 ± 320	21388.0 ± 70	24.0 ± 0.1	23.8 ± 0.1	397 ± 1	392 ± 6
Anc02	22896.0 ± 590	22394.0 ± 450	24.2 ± 0.1	24.0 ± 0.1	385 ± 0	385 ± 7
